# Unexpected Role of the Steroid-Deficiency Protein Ecdysoneless in Pre-mRNA Splicing

**DOI:** 10.1371/journal.pgen.1004287

**Published:** 2014-04-10

**Authors:** Ann-Katrin Claudius, Patrizia Romani, Tobias Lamkemeyer, Marek Jindra, Mirka Uhlirova

**Affiliations:** 1 Institute for Genetics and Cologne Excellence Cluster on Cellular Stress Responses in Aging-Associated Diseases (CECAD), University of Cologne, Cologne, Germany; 2 Dipartimento di Biologia Evoluzionistica Sperimentale, Università di Bologna, Bologna, Italy; 3 Proteomics Facility, CECAD, University of Cologne, Cologne, Germany; 4 Biology Center, Academy of Sciences of the Czech Republic, Ceske Budejovice, Czech Republic; University of Alberta, Canada

## Abstract

The steroid hormone ecdysone coordinates insect growth and development, directing the major postembryonic transition of forms, metamorphosis. The steroid-deficient *ecdysoneless^1^* (*ecd^1^*) strain of *Drosophila melanogaster* has long served to assess the impact of ecdysone on gene regulation, morphogenesis, or reproduction. However, *ecd* also exerts cell-autonomous effects independently of the hormone, and mammalian Ecd homologs have been implicated in cell cycle regulation and cancer. Why the *Drosophila ecd^1^* mutants lack ecdysone has not been resolved. Here, we show that in *Drosophila* cells, Ecd directly interacts with core components of the U5 snRNP spliceosomal complex, including the conserved Prp8 protein. In accord with a function in pre-mRNA splicing, Ecd and Prp8 are cell-autonomously required for survival of proliferating cells within the larval imaginal discs. In the steroidogenic prothoracic gland, loss of Ecd or Prp8 prevents splicing of a large intron from *CYP307A2/spookier* (*spok*) pre-mRNA, thus eliminating this essential ecdysone-biosynthetic enzyme and blocking the entry to metamorphosis. Human Ecd (hEcd) can substitute for its missing fly ortholog. When expressed in the Ecd-deficient prothoracic gland, hEcd re-establishes *spok* pre-mRNA splicing and protein expression, restoring ecdysone synthesis and normal development. Our work identifies Ecd as a novel pre-mRNA splicing factor whose function has been conserved in its human counterpart. Whether the role of mammalian Ecd in cancer involves pre-mRNA splicing remains to be discovered.

## Author Summary

Steroid hormones perform pivotal roles in animal development, sexual maturation, reproduction, and physiology. Also insects possess a hormonal steroid, commonly known as ecdysone, that was originally found to promote ecdyses in growing larvae and their metamorphosis to adults. Since the discovery of ecdysone-inducible puffs on the polytene chromosomes in the 1960's, genetics of the *Drosophila* fruit flies has substantially advanced our understanding of steroid hormone impact on gene regulation during development. In the present study, we have solved an old puzzle of the “*ecdysoneless*” mutant that has traditionally provided investigators with a steroid-deficient animal model. Unexpectedly, we find that the Ecdysoneless protein (Ecd) does not primarily regulate ecdysone biosynthesis but that a critical steroidogenic enzyme requires Ecd for splicing of its precursor mRNA (pre-mRNA). Ecd physically contacts the immensely complex pre-mRNA splicing machinery. Outside the ecdysone-producing gland, Ecd is essential for survival of dividing cells within developing tissues. Despite vast evolutionary distance, a human homolog of Ecd can functionally substitute for its counterpart in the fly. A conserved role of Ecd in pre-mRNA splicing might underlie a recently described involvement of mammalian Ecd in cell cycle progression and its contribution to malignancy of certain tumor types.

## Introduction

The insect steroid hormones, ecdysteroids, regulate growth, stimulate molting, and orchestrate tissues to undergo complex morphogenetic changes during metamorphosis [1]–[3]. Temporal control of ecdysteroid synthesis by the larval prothoracic gland (PG) is therefore critical (for recent reviews see [4], [5]). The biosynthetic pathway commences by converting cholesterol to 7-dehydrocholesterol by a Rieske oxygenase Neverland (Nvd) [6]. A short-chain dehydrogenase/reductase encoded by *shroud* (*sro*) [7] and cytochrome P450 (CYP) enzymes encoded by *spook* (*spo*)/*spookier* (*spok*), *phantom* (*phm*), *disembodied* (*dib*), and *shadow* (*sad*) then catalyze the subsequent steps to produce ecdysone (E) [8], [9]. Once released from the PG, E becomes hydroxylated in peripheral tissues by another CYP, Shade (Shd), to yield the main active hormone, 20-hydroxyecdysone (20E) [10]. Reflecting the necessity of 20E for early cuticle formation, *Drosophila melanogaster* loss-of-function mutants that are available for *sro* [7], *spo*, *phm*, *dib*, *sad*, and *shd* [8] die as embryos.

Understandably, *Drosophila* mutants that display reduced steroid titers during larval development provide invaluable experimental tools. Among these, the *ecdysoneless^1^* (*ecd^1^*) mutants are homozygous viable at 22°C, but exposure to 29°C reduces their ecdysteroid titer and causes a developmental arrest [11], [12]. The *ecd^1^* allele has been widely used since its discovery in 1977 [11] to test effects of ecdysteroid signaling on a number of processes from morphogenesis to reproduction to behavior. Yet why these mutants lack the hormone has not been determined. Our original identification of the *ecd* gene [13] has revealed homology from fission yeast to humans but none that would illuminate the mode of Ecd action.

Mammalian Ecd (also known as SGT1 and hEcd in humans) has been shown to stimulate cell proliferation by interacting with the Retinoblastoma (Rb) proteins [14]. Conditional deletion of the *Ecd* gene from mouse embryonic fibroblasts stalls these cells at the G_1_-S phase transition, suggesting that Ecd normally lifts the inhibitory effect of Rb on E2F-dependent cell cycle progression [14]. High hEcd expression has been correlated with malignancy of human breast [15] and pancreatic [16] tumors. In a mouse model, Ecd has been suggested to promote tumorigenesis via enhancing glucose import and glycolysis in the pancreatic tumor cells [16]. These observations illustrate the emerging importance of Ecd, but an underlying mechanism for Ecd action is still lacking.

A systematic mapping of *Drosophila* protein-protein interactions [17] has uncovered contacts between Ecd and proteins responsible for pre-mRNA splicing. Among these are members of the U5 small nuclear ribonucleoprotein particle (snRNP) complex, including orthologs of the RNA helicase Brr2, the GTPase Snu114, the Aar2 protein, and the highly conserved spliceosome core component, Prp8 [18]. Another global proteomic study [19] has detected a corresponding interaction between human Prp8 and hEcd. The spliceosome is a most elaborate machinery and pre-mRNA splicing is extensively coupled with transcription as well as with posttranscriptional mRNA surveillance and degradation of incorrectly spliced transcripts [20]. This network involves hundreds of proteins whose individual functions are often inferred from work on yeast, or still remain unknown [21]–[23]. Genetic studies in *Drosophila* have shown essential roles of several spliceosome components such as Prp8, Prp38, Prp31, or BCAS2 for tissue growth, cell proliferation or cell viability [24]–[27]. Proteomic dissection of spliceosomal complexes has revealed a large overlap in protein composition between *Drosophila* and humans while suggesting novel and/or fly-specific components [28].

With the hypothesis that Ecd might be a new player in pre-mRNA splicing, we have verified that Ecd interacts with a complex containing *Drosophila* Prp8, Aar2, Brr2, and Snu114 orthologs. Consistently with a vital role in pre-mRNA splicing, we show that Ecd is required by dividing imaginal disc cells for survival, even when their apoptosis is blocked or cell growth enhanced. In the larval PG, loss of Ecd compromises pre-mRNA splicing and abolishes protein expression of the essential steroidogenic enzyme Spok (CYP307A2), thus accounting for the systemic steroid deficiency in *ecd* mutants. Remarkably, human Ecd can functionally substitute for its fly ortholog.

## Results

### Ecd binds proteins within the spliceosomal U5 snRNP complex

**Figure 1:**
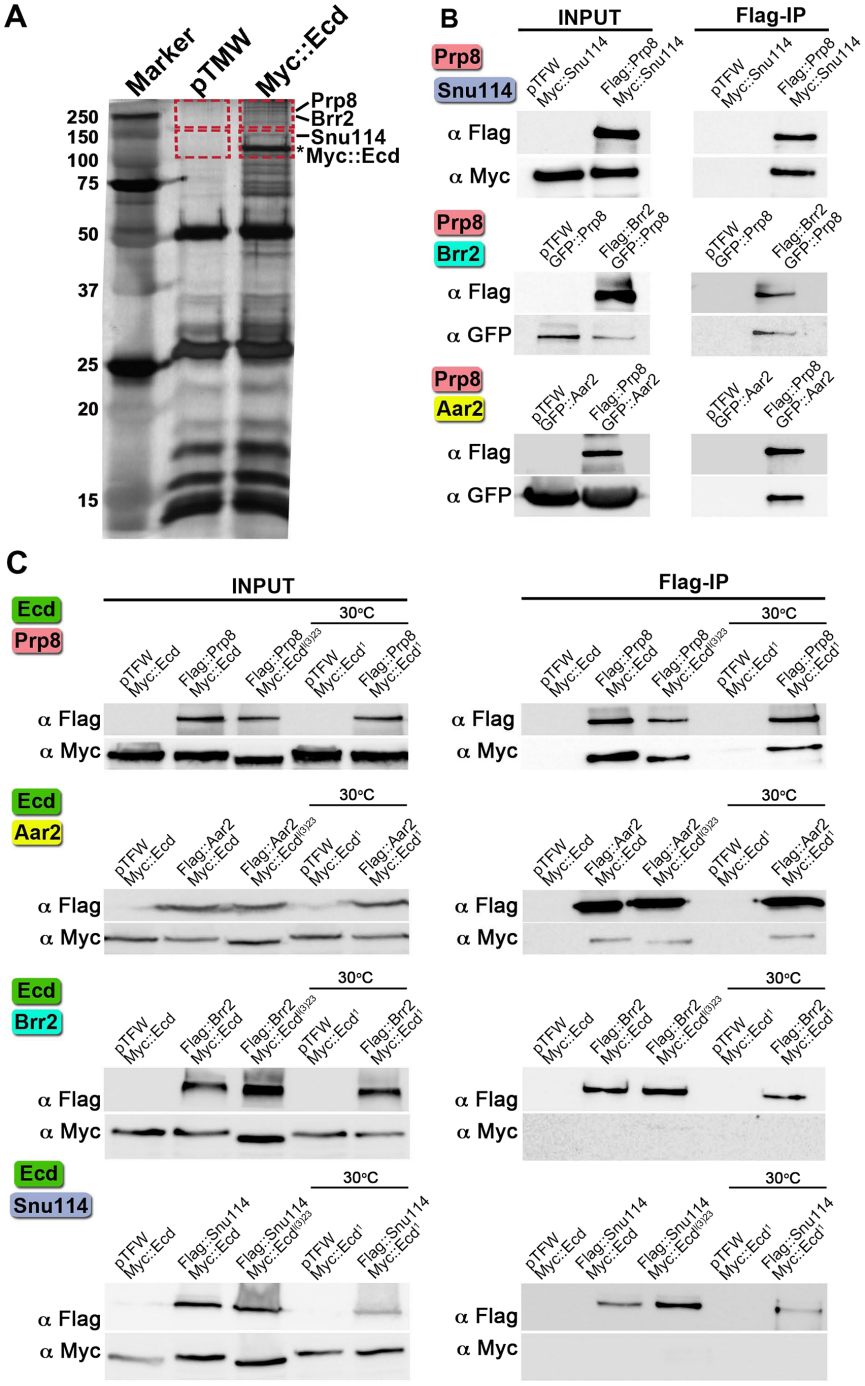
Interactions of Ecd with proteins of the U5 snRNP complex. (A) Extracts from *Drosophila* S2 cells expressing the Myc::Ecd fusion protein or the Myc tag alone (pTMW) were affinity purified using the Myc epitope and resolved on silver-stained SDS-PAGE. Mass spectrometry on the gel sections (indicated by rectangles) identified Prp8, Brr2, and Snu114 proteins associated with Myc::Ecd (asterisk). (B) *Drosophila* Prp8 interacts with orthologs of known spliceosomal proteins: Snu114, Brr2, and Aar2. (C) Flag-tagged Prp8 and Aar2 but not Brr2 and Snu114 proteins bound Ecd or its mutant variants as assessed by Flag immunoprecipitation (Flag-IP). Cells expressing the temperature-sensitive Ecd^1^ mutant were upshifted to 30°C 45 min prior to lysis. In all assays (B, C), the pairs of proteins were expressed in S2 cells and their epitope tags were used for Flag-IP and immunoblot detection as indicated. The empty pTFW vector expressing the Flag peptide alone was included in control assays.

The *Drosophila* Ecd protein has been recently associated with the U5 snRNP complex [17]. In order to verify the proteome-wide data, we expressed Myc epitope-tagged Ecd in *Drosophila* S2 cells and using mass spectrometry we examined material that co-precipitated with Myc::Ecd (Figure 1A). This analysis identified three proteins of the U5 snRNP complex that were previously shown to associate with Ecd [17], namely orthologs of the budding yeast (*Saccharomyces cerevisiae*) Prp8p, Snu114p, and Brr2p that are encoded by *D. melanogaster* genes *CG8877*/*prp8*, *CG4849/eftud2*, and *CG5931*/*l(3)72Ab*, respectively. Our mass spectrometry did not detect another Ecd interactor, identified as a product of the *CG12320* gene [17] that is homologous to *S. cerevisiae* Aar2p. For clarity, we will refer to the *Drosophila* proteins as Prp8, Snu114, Brr2, and Aar2.

To test the individual protein-protein interactions, we cloned the above *Drosophila* genes and performed co-immunoprecipitation with pairs of their epitope-tagged products expressed in S2 cells. First, we verified interactions between the fly counterparts of the known spliceosomal components. As expected, binding occurred between Prp8 and Snu114, Prp8 and Brr2, and Prp8 and Aar2 (Figure 1B). Of these four proteins, Prp8 and Aar2 formed complexes with Ecd, whereas binding between Ecd and either Brr2 or Snu114 was not detected (Figure 1C). It was of interest to examine interactions of mutated Ecd versions that occur in *Drosophila*. We chose the temperature-sensitive *ecd^1^* allele that carries a single Pro-656 to serine substitution and the non-conditional lethal, *ecd^l(3)23^*, where the Ecd polypeptide is prematurely terminated after Ser-649, thus lacking the C-terminal 35 amino acids [13]. Both the Ecd^1^ (tested at a non-permissive temperature of 30°C) and Ecd^l(3)23^ mutant proteins retained the capacity to bind Prp8 and Aar2 (Figure 1C). This indicates that the steroid-deficiency and lethality phenotypes in *Drosophila* result from a failure of the mutant Ecd protein to perform functions other than binding Prp8 and Aar2. The above results suggest that Ecd interacts with at least two well-established members of the U5 snRNP complex, of which Prp8 is evolutionarily the best-conserved core protein of the spliceosome [18].

### Subcellular localization of the spliceosomal proteins and Ecd

**Figure 2:**
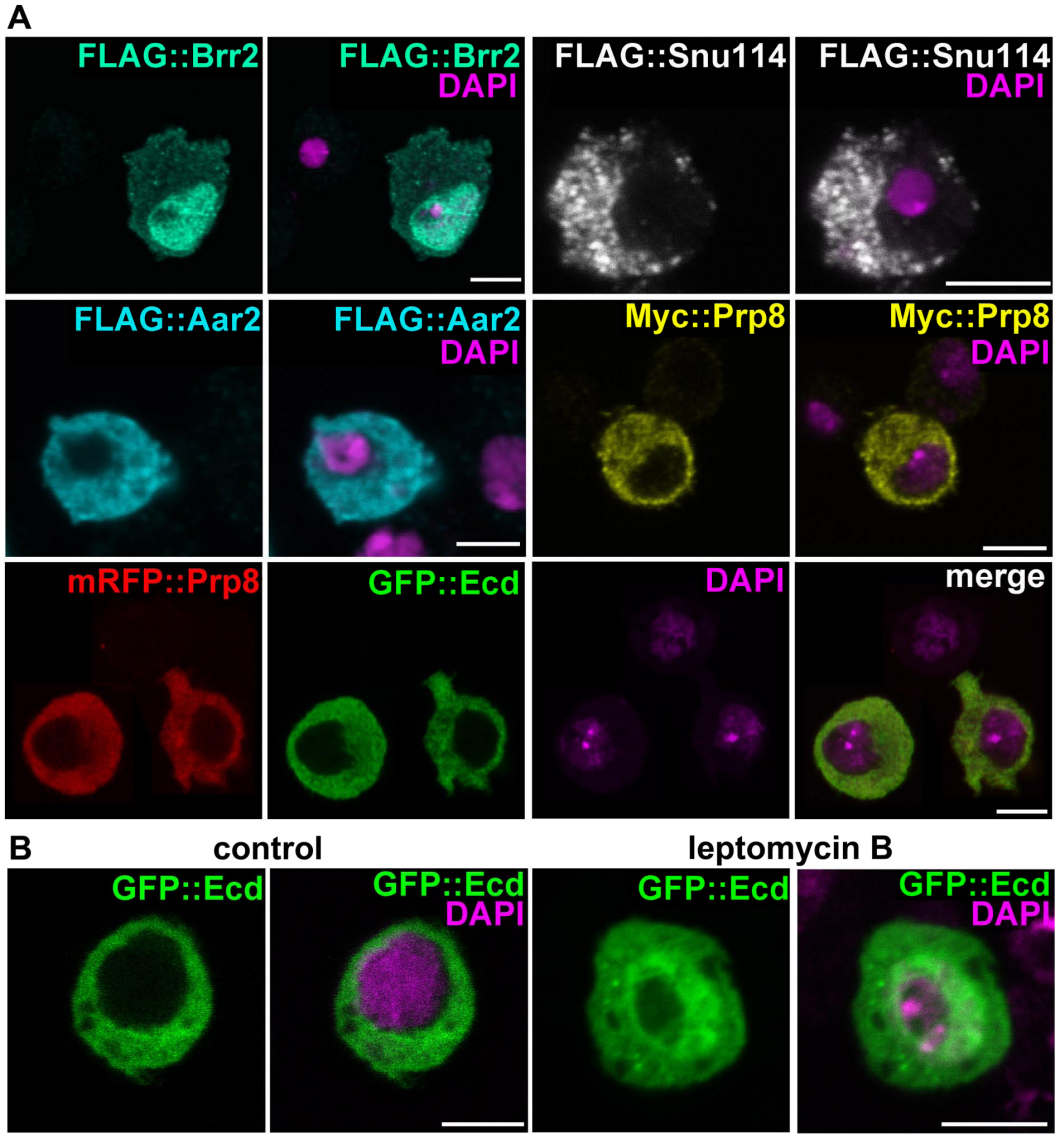
Subcellular localization of Ecd and the U5 snRNP proteins. (A) Brr2 was enriched in cell nuclei while Ecd, Prp8, Snu114, and Aar2 were detected in the cytoplasm. The Flag and Myc epitope-tagged proteins were expressed in S2 cells; mRFP::Prp8 and GFP::Ecd (bottom row) were co-transfected. (B) Inhibition of nuclear export with leptomycin B resulted in nuclear retention of the GFP::Ecd protein. Nuclei are stained with DAPI. All images are single confocal sections. Scale bars, 5 µm.

In *S. cerevisiae*, a part of the U5 snRNP complex that contains Prp8p, Snu114p, and Aar2p assembles in the cytoplasm before being imported to the nucleus, where Brr2p replaces Aar2p [29], [30]. Localization of *Drosophila* Prp8, Snu114, Aar2 or Brr2 has not been previously reported. Using the epitope-tagged proteins in transfected S2 cells, we detected Prp8, Snu114, and Aar2 predominantly in the cytoplasm, whereas Brr2 occurred in the nucleus (Figure 2A). Expression of Flag::Prp8 in transgenic *Drosophila* larvae revealed that Prp8 also resided in the cytoplasm of the PG (Figure S1A).

The Ecd protein was previously detected in the cytoplasm of *Drosophila* PG cells [13]. We confirmed this result with a new antibody raised against the N-terminal part of Ecd for the endogenous protein (Figure S1B, S1C) and for Ecd overexpressed in the PG using the *phm-Gal4* driver (Figure S1D). This antibody also detected a clear cytoplasmic signal in the wing imaginal discs (Figure S2). The cytoplasmic localization of hEcd in human cells was shown to depend on leptomycin B-sensitive nuclear export [31]. In transfected S2 cells, GFP-tagged Ecd co-localized in the cytoplasm with Prp8 (Figure 2A). Only when the cells were treated with leptomycin B, some of the GFP::Ecd fusion protein was retained in the nucleus (Figure 2B). Therefore, together with the spliceosomal proteins with which Ecd interacts, Ecd primarily resides in the cytoplasm. Like its human counterpart, Ecd is subject to active nuclear export.

### Cell-autonomous requirement for Ecd and Prp8 in imaginal discs

**Figure 3:**
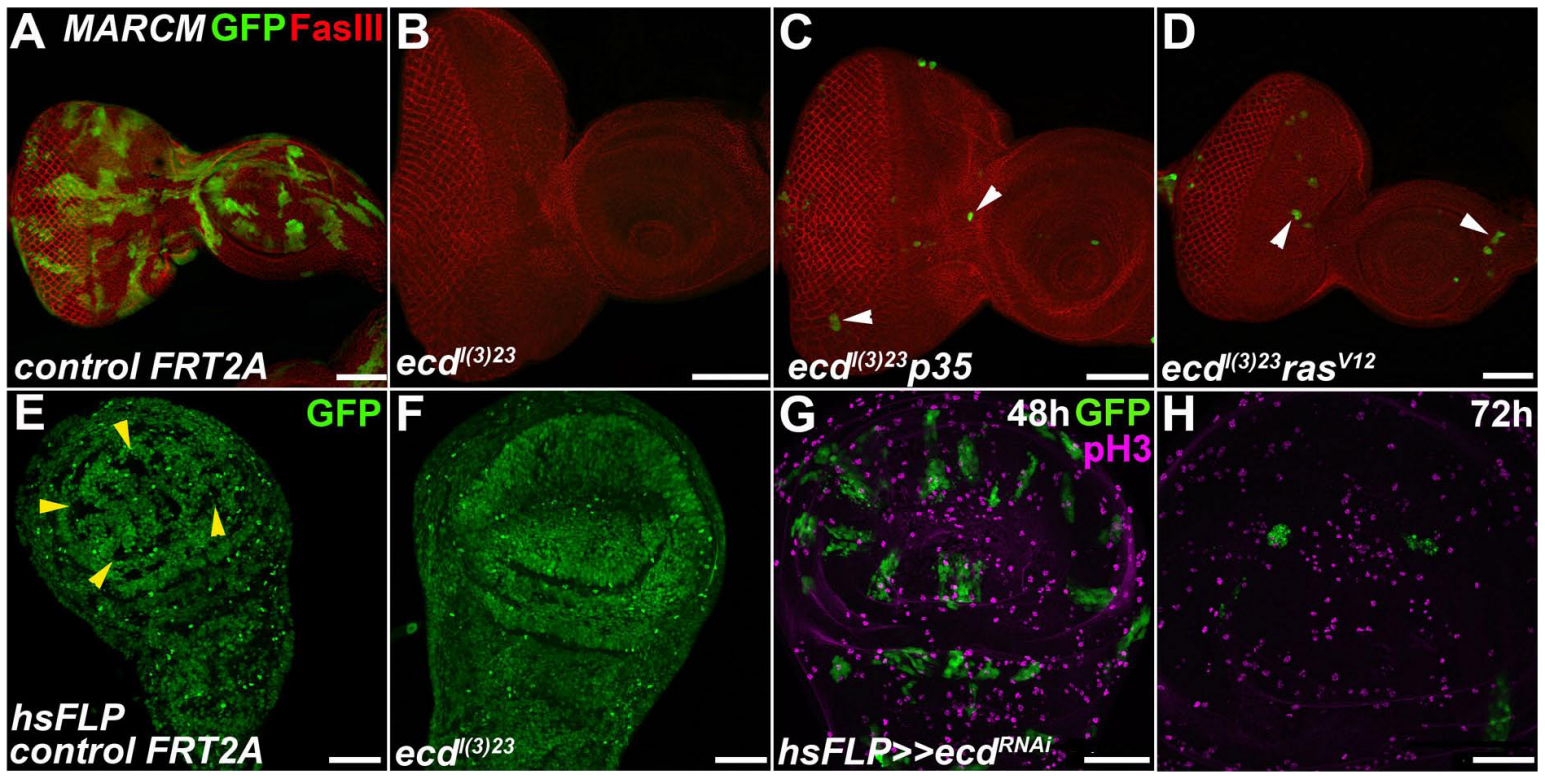
Ecd is required for survival of imaginal disc cells. (A–F) In contrast to abundant, sizable control clones (green in A and GFP-negative in E, yellow arrowheads), *ecd^l(3)23^* homozygous mutant clones could be recovered neither in eye/antennal (B) nor wing (F) imaginal discs. Simultaneous inhibition of apoptosis with a pan-caspase inhibitor p35 (*ecd^l(3)^* ^23^ *p35*) (C) or cell growth enhancement with activated Ras^V12^ (*ecd^l(3)^* ^23^ *ras^V12^*) (D) yielded rare and extremely small clones in eye/antennal discs (white arrowheads). Fasciclin III (FasIII) staining of lateral cell membranes reveals the overall tissue morphology. (G–H) *ecd^RNAi^* clones (green) were eliminated from the wing imaginal epithelium within 72 h after induction using the heat-shock flip-out technique. Only rare *ecd^RNAi^* cells labeled phospho-histone 3 (pH3) positive relative to many mitotically active surrounding control cells. Scale bars, 50 µm.

If Ecd plays a role in pre-mRNA splicing as suggested by its protein interactions, its loss would be expected to disturb vital cellular functions as has been found for several *Drosophila* spliceosomal proteins including Prp8 [24]–[27]. To track the fate of Ecd-deficient imaginal cells *in vivo*, we employed the MARCM technique [32] using the non-conditional *ecd^l(3)23^* allele that behaves as a genetic null [13]. In contrast to control, no mitotic clones homozygous for this mutation were found in eye/antennal or wing imaginal discs of third-instar larvae (Figure 3A, 3B, 3E, 3F). Rare and extremely small *ecd^l(3)23^* clones could be detected when cell death was prevented in these clones with the anti-apoptotic protein p35, or when their cellular growth was enhanced with activated Ras^V12^ (Figure 3C, 3D). To achieve a milder loss-of-function effect, we generated transgenic flies for RNAi-mediated silencing of *ecd*. Observation of wing discs two days after heat-dependent clonal induction of *ecd* dsRNA revealed frequent *ecd^RNAi^* clones. These gradually disappeared over the next 24 hours (Figure 3G, 3H), indicating that the cells autonomously required Ecd to survive.

**Figure 4:**
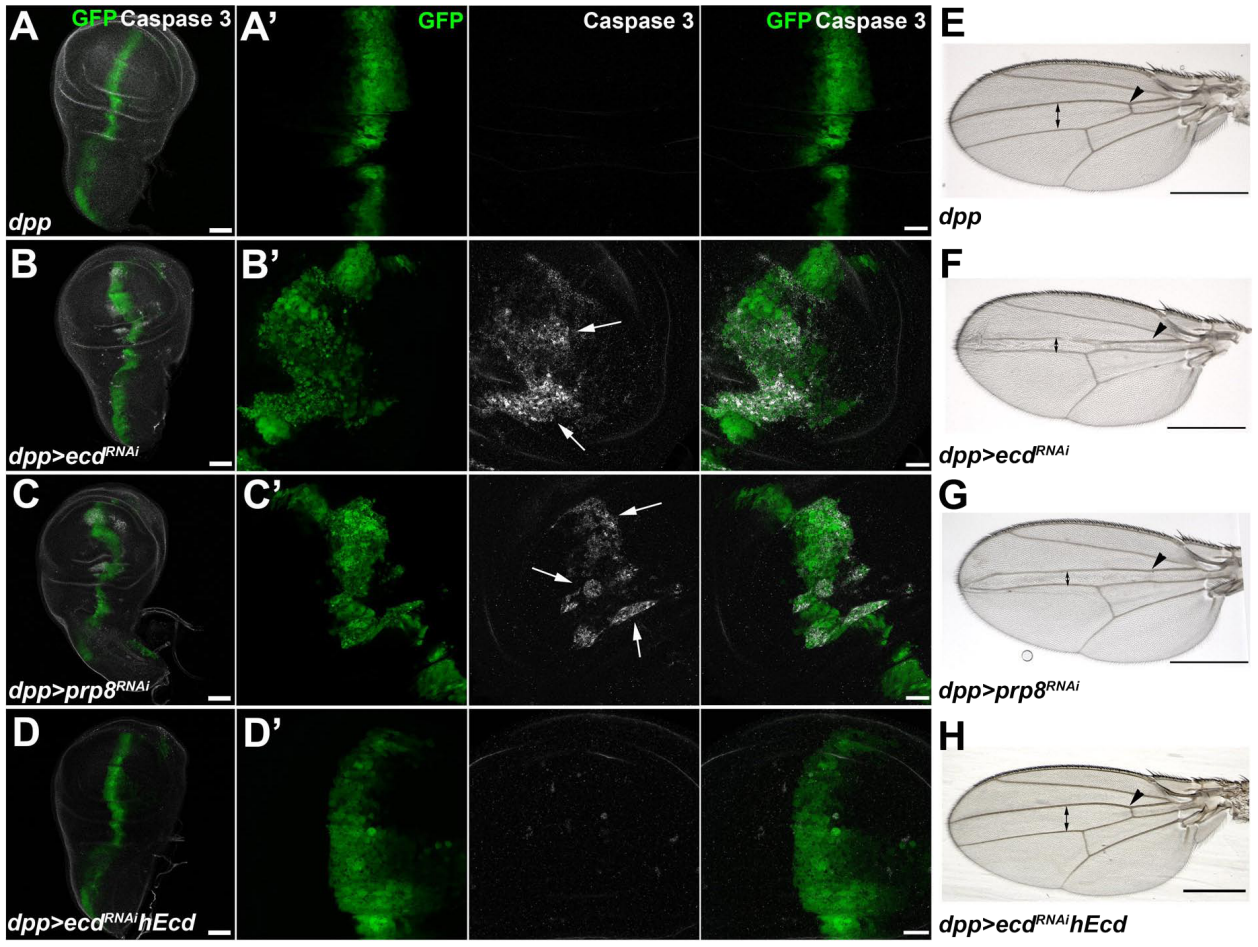
Depletion of Ecd or Prp8 causes apoptotic phenotypes in developing wings. (A–D) RNAi knockdown of *ecd* (B) and *prp8* (C) induced with the *dpp-Gal4* driver disrupted the regular pattern of the *dpp* expression domain (visualized by co-expression of *GFP*) in third-instar wing imaginal discs and caused appearance of apoptotic, Caspase 3 positive cells (B′, C′, white arrows). Expression of human Ecd (hEcd) averted the apoptotic phenotype caused by *ecd* RNAi (D, D′). (E–H) The intervein region corresponding to the *dpp* expression domain was reduced (double arrows) and the anterior crossvein (arrowhead) was lost in the wings of *dpp>ecd^RNAi^* (F) and *dpp>prp8^RNAi^* (G) adult flies. The *ecd* RNAi phenotype was rescued by expression of hEcd (H). Images in (A–D) are maximum-intensity projections of multiple confocal sections; panels A′ through D′ are higher-magnification, single sections from the corresponding images in (A–D). Scale bars are 50 µm (A–D), 20 µm (A′–D′), and 1 mm (E–H).

To see the effect of Ecd knockdown on morphogenesis, we induced *ecd* RNAi in a restricted region along the anterior-posterior boundary of the wing imaginal disc using the *dpp-Gal4* driver. Figure S2 shows depletion of the Ecd protein from the *dpp*-expressing cells. The loss of Ecd disrupted the regular pattern of the *dpp* expression domain, and the affected cells underwent apoptosis as assessed by active Caspase 3 staining (Figure 4A, 4B). Consequently, the adult wings of *dpp>ecd^RNAi^* flies displayed reduced size of specific intervein regions and loss of the anterior crossvein (Figure 4E, 4F). The same anomalies were induced by RNAi against the components of the U5 snRNP complex, Prp8 (Figure 4C, 4G) and Brr2 (Figure S3A, S3B). Overexpression of Prp8 in the Ecd-deficient cells did not suppress the *ecd* RNAi phenotype (Figure S3C, S3D), suggesting that Ecd plays a unique role that cannot be substituted by surplus of its partner protein. Strikingly, apoptosis of imaginal disc cells expressing *ecd* dsRNA as well as the adult wing defect could be averted by supplementing the human ortholog, hEcd (Figure 4D, 4H).

### Loss of Ecd disrupts splicing of *spok* pre-mRNA and eliminates its protein product

**Figure 5:**
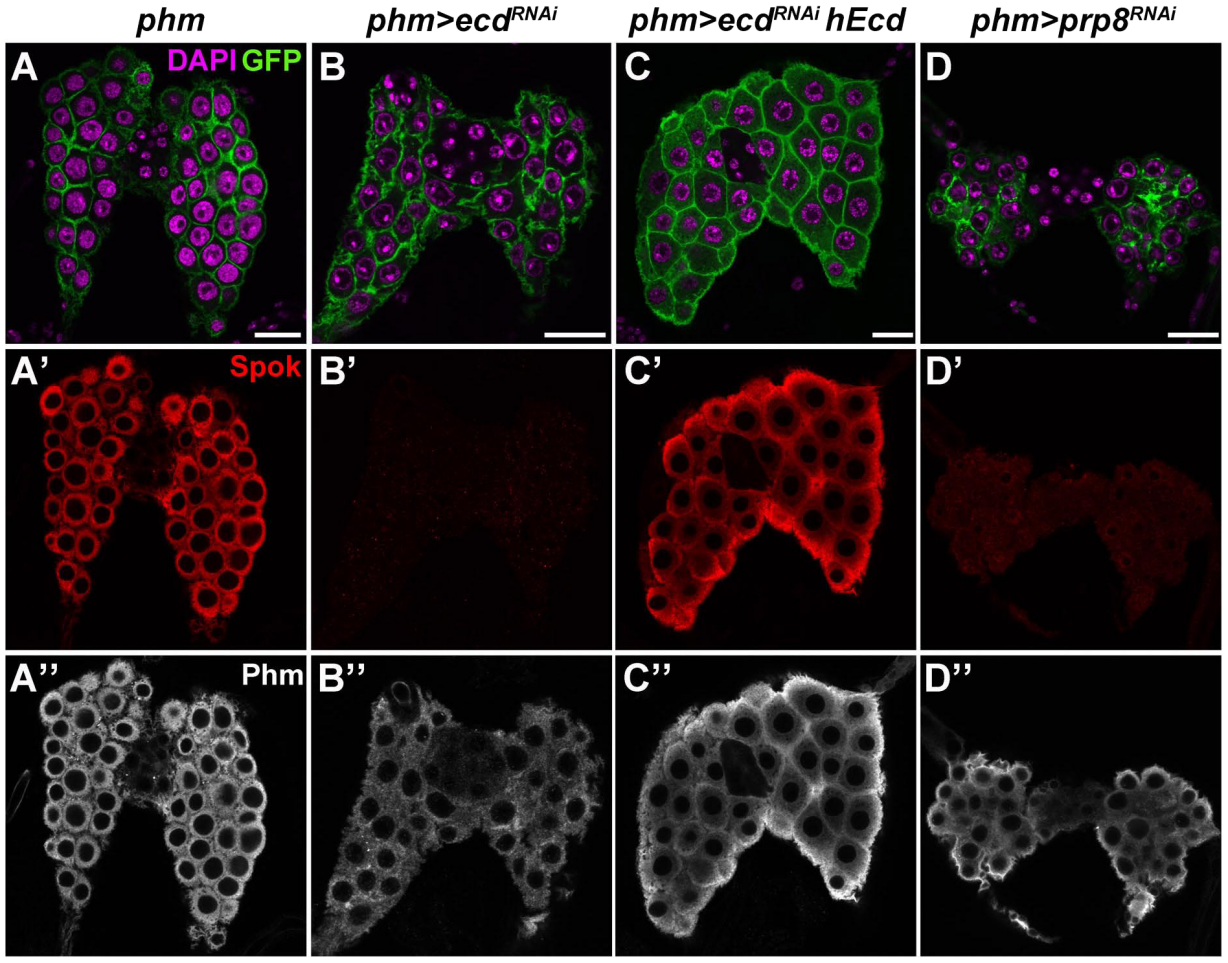
Ecd and Prp8 are required for expression of Spok in the PG. (A–D) Relative to control PG dissected 6 days AEL (A′, A″), expression of the Spok protein (B′) was undetected while the Phm signal (B″) was weakened in the PG of *phm>ecd^RNAi^* (B) and *phm>prp8^RNAi^* (D′, D″) larvae. Note the moderate reduction in size of PG cells and nuclei in *phm>ecd^RNAi^* (B) compared to a more severe PG deterioration in *phm>prp8^RNAi^* larvae (D). Expression of hEcd restored Spok and Phm expression (C′, C″) and improved the morphology of the Ecd-deficient PG (C). Cell membranes are decorated with CD8::GFP; DAPI stains the nuclei. Panels show single confocal sections. Scale bars, 20 µm. See Figure S1 for RNAi-mediated depletion of Ecd.

To address the putative role of Ecd in ecdysone biosynthesis, we removed the Ecd protein specifically from the PG by triggering *ecd* RNAi with the *phm-Gal4* driver [33] (Figure S1C). Affected larvae reached the third instar but were unable to pupate. Unlike in the imaginal discs, depletion of Ecd was not cell-lethal in the polyploid PG, although it reduced the size of PG cells and nuclei (Figure 5A, 5B). Using available antibodies, we could therefore examine expression of steroidogenic enzymes, namely CYP307A2/Spok and CYP306A1/Phm, in late-third instar *phm>ecd^RNAi^* larvae. The strong Spok staining in control PGs was completely lost in the Ecd-deficient gland, whereas the Phm signal appeared only partly reduced (Figure 5A, 5B). While both enzymes are indispensable for E biosynthesis, Spok acts upstream of Phm in the larval PG to mediate the essential conversion of 7-dehydrocholesterol to ketodiol [33], [34]. Thus, PGs deprived of Spok cannot synthesize ecdysone even in the presence of Phm. As was the case in the wing imaginal discs, expressing the human Ecd ortholog in the PG compensated for the depletion of the endogenous Ecd protein. hEcd improved morphology of the gland (Figure 5C) and restored expression of Spok (Figure 5C′).

**Figure 6:**
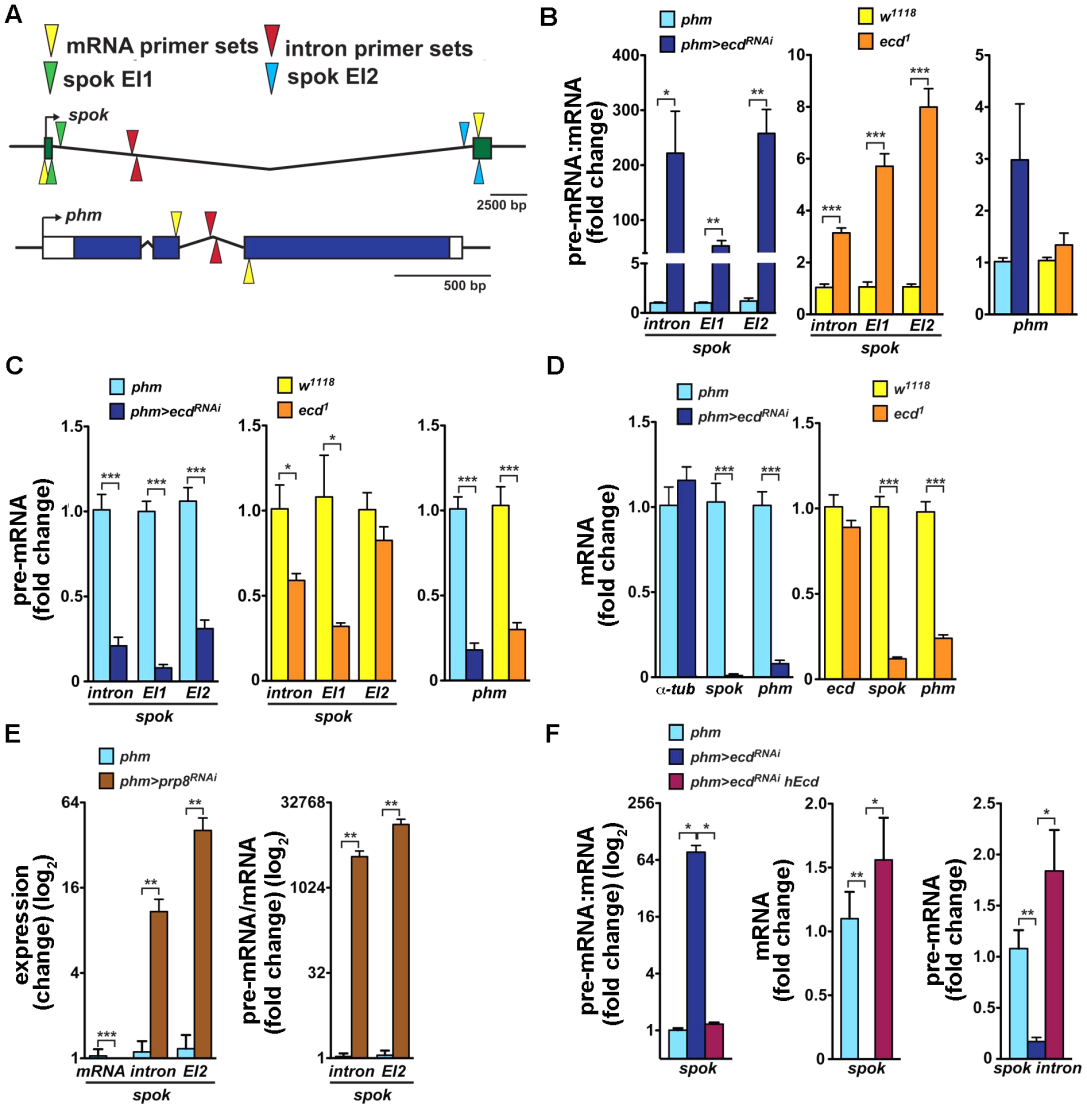
Loss of Ecd interferes with splicing of *spok* pre-mRNA. (A) Schematic of *Drosophila spok* and *phm* gene loci. Open and colored boxes represent untranslated and translated exons, respectively; black arrows point in the direction of transcription. Colored arrowheads mark the positions of primer pairs used to discriminate between pre-mRNA and mRNA species by qRT-PCR: yellow, in exons separated by an intron; red, within an intron; green and blue, spanning exon-intron boundaries of the first (EI1) and second (EI2) *spok* exon, respectively. (B) Pre-mRNA:mRNA ratios, determined by qRT-PCR with primers depicted in (A), were significantly elevated for *spok* but not for *phm* in third-instar *phm>ecd^RNAi^* larvae and in *ecd^1^* mutants at 29°C. (C, D) *spok* and *phm* pre-mRNA (C) and mRNA (D) levels decreased in *phm>ecd^RNAi^* and *ecd^1^* larvae. Levels of *α-tubulin 84B* mRNA did not change upon *ecd* RNAi and expression of *ecd* itself was unaffected by the *ecd^1^* mutation (D). (E) RNAi knockdown of *prp8* in the PG diminished *spok* mRNA, whereas unspliced transcripts accumulated, causing the pre-mRNA:mRNA ratio to rise dramatically. (F) Expression of hEcd in the Ecd-deficient PG restored normal *spok* transcription and pre-mRNA splicing as judged from restored levels of pre-mRNA, mRNA, and their ratio. In all experiments, qRT-PCR was performed with total RNA from whole larvae 6 days AEL, and levels of *rp49* transcripts were used for normalization. The pre-mRNA:mRNA ratios in (B, E, F) were calculated from the normalized qRT-PCR data by dividing values obtained with intron primer sets (A, red triangles) or primers spanning exon-intron boundaries (green or blue triangles) with values obtained using mRNA-specific primers (yellow triangles). Data are mean ± S.E.M; *n*≥4; *p<0.05, **p<0.01, and ***p<0.001.

Similarly to *ecd* RNAi, knockdown of the spliceosomal proteins Prp8 (Figure 5D) and Brr2 (Figure S4A, S4B) also reduced size of the PG and eliminated the Spok protein while only partially reducing Phm levels. These results suggested that expression of Spok, but not of Phm, was highly sensitive to perturbation of pre-mRNA splicing. Interestingly, the *phm* gene contains two small introns, whereas *spok* lies within the heterochromatin and its single intron separates the two coding exons by nearly 30 kilobases (Flybase, http://flybase.org/) (Figure 6A).

To examine whether the absence of Ecd affects *spok* pre-mRNA splicing, we determined the pre-mRNA:mRNA ratio, a standard measure of intron retention or splicing defect (e.g., [35]–[37]), in *phm>ecd^RNAi^* larvae and in larvae homozygous for the temperature-sensitive *ecd^1^* allele. mRNA levels were measured using quantitative reverse-transcription (qRT) PCR with primers positioned in two separate exons, and pre-mRNA was amplified with primer pairs spanning exon-intron boundaries or primers within the intron (Figure 6A). All RNA samples were free of residual genomic DNA and qRT-PCR data were normalized to *rp49* mRNA levels that did not change appreciably between control and *ecd*-deficient larvae (Figure S5). Because *spok* and *phm* are expressed specifically in the PG during larval development, we were able to use RNA from entire larvae.

As assessed with all three pre-mRNA-specific primer sets, *spok* pre-mRNA:mRNA ratio strongly increased in both *phm>ecd^RNAi^* larvae and in *ecd^1^* mutants upshifted to 29°C (Figure 6B). The relative enrichment of unspliced to spliced *spok* transcript was markedly stronger upon PG-specific knockdown of *ecd* than in the hypomorph mutants. Unexpectedly, loss of *ecd* also reduced levels of *spok* pre-mRNA, again more substantially in *phm>ecd^RNAi^* larvae (Figure 6C), suggesting that in addition to splicing, either *spok* transcription or stability of its pre-mRNA might have been affected. Consistently with the absence of the Spok protein (Figure 5B′), *ecd* RNAi diminished *spok* mRNA (Figure 6D). Levels of *phm* pre-mRNA and mRNA were also lowered in PGs of *ecd*-deficient animals relative to controls (Figure 6C, 6D). However, in contrast to *spok* there was no significant increase of the pre-mRNA:mRNA ratio and therefore no appreciable intron retention in the case of *phm* (Figure 6B). These data suggested that Ecd was required for splicing of the large intron from the *spok* pre-mRNA. For comparison, we examined the effect of Prp8 knockdown. As expected, *spok* mRNA disappeared in *phm>prp8^RNAi^* larvae due to compromised splicing (Figure 6E). However, because the absolute amount of unspliced *spok* transcript accumulated upon *prp8* RNAi, the pre-mRNA:mRNA ratio rose dramatically more than in the case of *ecd* RNAi (Figure 6E).

Finally, we tested whether human Ecd restored Spok expression at the level of pre-mRNA splicing. In a separate set of experiments, we confirmed that the loss of *spok* mRNA was accompanied by intron retention (increased pre-mRNA:mRNA ratio, Figure 6F). Importantly, expression of hEcd in the PG of *phm>ecd^RNAi^* larvae restored levels of *spok* mRNA, pre-mRNA, and of their normal proportion (Figure 6F), suggesting that the human protein could substitute for Ecd in splicing of *spok* pre-mRNA.

### Systemic effect of PG-specific loss of Ecd

**Figure 7:**
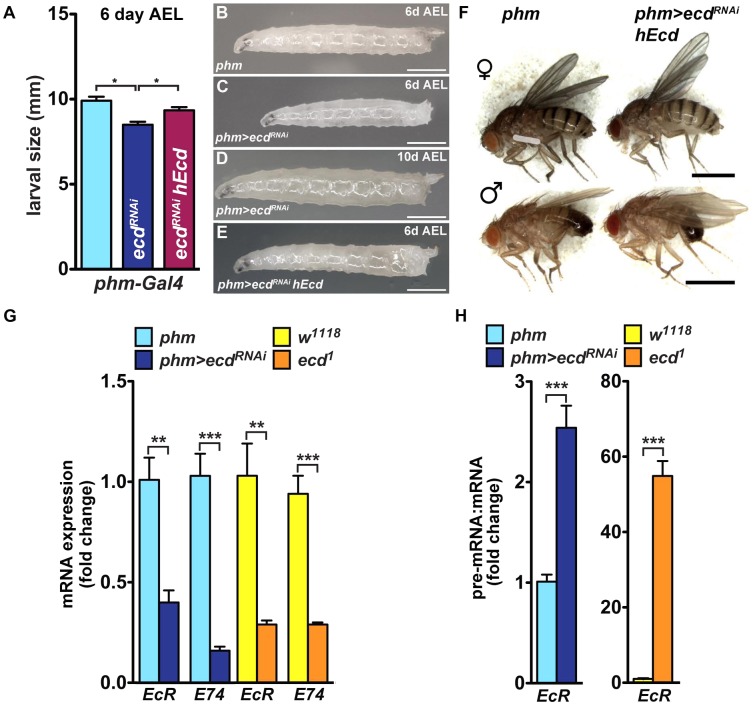
PG-specific loss of Ecd results in systemic effects. (A–E) Growth of *phm>ecd^RNAi^* larvae was retarded. Although reaching the third instar, *phm>ecd^RNAi^* larvae were smaller on day 6 AEL (A, C) compared to controls (B) or larvae supplemented with hEcd (*phm>ecd^RNAi^ hEcd*) (E). Deficiency of Ecd in the PG prevented metamorphosis, generating permanent, eventually lethal larvae (D). (F) Expression of hEcd in the Ecd-deficient PG restored development of normally sized adults. (G) Expression of *EcR* and *E74* (as assessed by qRT-PCR with primer sets detecting all alternatively spliced mRNA isoforms of each gene, see Table S1) was lowered to a similar degree in *phm>ecd^RNAi^* and in *ecd^1^* larvae at 29°C. (H) The *EcR* pre-mRNA:mRNA ratio increased much more dramatically in *ecd^1^* mutants at 29°C than in *phm>ecd^RNAi^* larvae. Data are mean ± S.E.M; *n*≥4; **p<0.01, and ***p<0.001.

Because the PG produces a circulating hormone, knockdown of Ecd would be expected to affect the entire organism even when restricted to the gland. In agreement with the absence of the Spok/CYP307A2 protein (Figure 5B′), third-instar *phm>ecd^RNAi^* larvae on day 6 after egg laying (AEL) showed reduced ecdysteroid titer (Figure S6A). Compared to controls, they were retarded in growth (Figure 7A–C). Without interruption, they continued to feed for another 10 or more days, producing large “permanent” larvae (Figure 7D). This phenotype corresponded with effects of lacking ecdysteroid surge and inadequate expression of steroidogenic genes including *spok* and *phm* [38], [39]. Although feeding *phm>ecd^RNAi^* larvae on day 5 AEL with 20E was not sufficient for pupation, it induced wandering behavior (Figure S6B). Importantly, substituting hEcd for the depleted fly protein in the PG increased ecdysteroid titer (Figure S6A) and restored normal growth and development of *phm>ecd^RNAi^* animals to adults (Figure 7A, 7E, 7F).

The PG-specific knockdown of Ecd was accompanied by lowered mRNA levels of at least two 20E-response genes, the *Ecdysone receptor* (*EcR*) and *E74*, as assessed in whole *phm>ecd^RNAi^* larvae (Figure 7G). A similar reduction in *EcR* and *E74* mRNAs occurred in *ecd^1^* mutant larvae at 29°C (Figure 7G). However, *ecd^1^* mutants could have lost these transcripts for two different, mutually non-exclusive reasons: (1) due to the lack of circulating ecdysteroids, and/or (2) because Ecd may be required for splicing of the *EcR* and *E74* pre-mRNAs, both of which contain multiple large introns. Indeed, we found a major rise in the pre-mRNA:mRNA ratio for *EcR* (but not for *E74*; data not shown) in *ecd^1^* mutants, and a much less pronounced increase of this ratio in *phm>ecd^RNAi^* larvae (Figure 7H). These data suggest that *EcR* pre-mRNA splicing was sensitive to loss of *ecd* function in tissues throughout the body. In support of this notion, we observed that dietary 20E did not induce *EcR* mRNA expression in *ecd^1^* mutants at 29°C nearly as efficiently as under the permissive temperature or in *phm>ecd^RNAi^* larvae (Figure S6C), where *ecd* function in the peripheral tissues was unaffected.

## Discussion

Ecd, originally discovered through the “ecdysoneless” *Drosophila ecd^1^* mutant [11], has become known as a product of the *CG5714* gene [13]. The steroid deficiency results from an autonomous failure of the larval PG [12], [40] and of the adult ovary [11], [40], [41]. However, some effects of the *ecd^1^* allele are autonomous to the imaginal discs and other tissues, and therefore not attributable to the lack of the hormone [41], [42]. This notion has been confirmed upon molecular definition of *ecd*-null mutations whose effects could not be remedied with exogenous 20E [13]. As no causal relationship between Ecd and E biosynthesis could be established, Ecd has been omitted from the current list of ecdysteroidogenic factors [4], [5], [8], [9]. Nonetheless, the *ecd^1^* background is still in use to demonstrate specific effects of 20E (e.g., [43]).

Our present work shows that Ecd indeed does not primarily regulate steroidogenesis. Although abolished expression of the essential steroidogenic enzyme Spok in Ecd-deficient larvae inevitably causes ecdysone deficiency, Ecd should not be regarded as a regulator of the E biosynthetic pathway. Rather, the absence of Spok in the PG reflects a more general role of Ecd within the complex network of pre-mRNA splicing. This function is required in the PG as well as in some other *Drosophila* organs, and is likely to be common to flies and mammals.

### A role for Ecd in pre-mRNA splicing

A proteome-wide study [17] has uncovered interactions of *Drosophila* Ecd with multiple U5 snRNP-associated spliceosomal proteins. We likewise found a complex of Ecd with Prp8, Snu114, and Brr2 using mass spectrometry, and interactions of Ecd with Prp8 and Aar2 by immunoprecipitation. We detected Brr2 in cell nuclei, whereas Ecd co-localized with Prp8, Snu114, and Aar2 in the cytoplasm of *Drosophila* cells. In contrast, *S. cerevisiae* Prp8p resides in the yeast nuclei owing to its nuclear localization signal [29]. Although Prp8 is remarkably well conserved [18], this particular sequence shows poor homology with the fly protein. Nuclear import of *Drosophila* Prp8 might therefore rely on another mechanism. Nonetheless, our results conform to a current model from *S. cerevisiae*, where Prp8p, Snu114p, and Aar2p preassemble in the cytoplasm, then upon nuclear import Aar2p is replaced by Brr2p as the U5 snRNP complex matures [29], [30]. Nucleo-cytoplasmic shuttling of Ecd, which also occurs in human cells [31], corresponds with such translocation.

Phenotypes caused by disrupted pre-mRNA splicing match those inflicted by loss of Ecd. Cell death of *Drosophila* imaginal disc clones lacking spliceosomal proteins Prp38, MFAP1, and BCAS2 has been reported [24], [25]. Our data show that knockdown of the Prp8 and Brr2 proteins caused apoptosis of imaginal disc cells and recapitulated a specific *ecd* RNAi wing phenotype. Removal of Prp8 or Brr2 from the PG eliminated the Spok protein, confirming that its expression was sensitive to deficiency in pre-mRNA splicing factors. Similar to Prp8 knockdown, *spok* pre-mRNA:mRNA ratio strongly increased in *ecd^1^* or *phm>ecd^RNAi^* larvae, thus evidencing a role of Ecd in *spok* pre-mRNA splicing. However, removal of Ecd also affected transcription as judged from reduced levels of *spok* or *phm* pre-mRNAs. It is important to note here that pre-mRNA splicing is not an isolated event but that it is intimately coupled with transcriptional elongation [44], [45] as well as with 3′-end processing, quality control, and nuclear export or degradation of the RNA product (reviewed in [20]). Indeed, there are indications that hEcd may be involved in transcriptional regulation in mammalian cells [31].

An intriguing question is why would dysfunction of Ecd impact splicing of *spok* but not *phm* pre-mRNA in the steroid-producing gland? Surprisingly, even the relatively few and simple intron-containing genes in *S. cerevisiae* have been shown to respond differently to mutations in individual spliceosome core components [36]. In multicellular organisms, spliceosomal proteins such as Prp8 are not ubiquitously expressed and not equally required to splice every pre-mRNA [46]. Instead, many perform exquisitely tissue-specific, gene-specific, and even exon-specific functions. An RNAi screen in *Drosophila* cells has uncovered the necessity of several core spliceosomal proteins including Brr2 for inclusion of particular exons in different, alternatively spliced transcripts [47].

A striking example of how remarkably specific physiological functions may be affected by faulty pre-mRNA splicing is the human disease retinitis pigmentosa (RP), where degeneration of photoreceptor cells leads to blindness. Autosomal-dominant forms of RP are linked to mutations in Prp8, Prp31, Prp3, and PAP1 homologs that contribute to the U5•U4/U6 tri-snRNP complex [46], [48]. One plausible explanation for the sensitivity of the photoreceptor neurons to defective splicing is insufficient production of mRNAs that are highly expressed in these cells [48]. It has been argued that ubiquitously expressed pre-mRNAs carry universal splicing signals in order to be correctly spliced in any cell type, whereas transcripts with restricted expression patterns might rely on less robust, tissue-specific splicing signals that are more prone to fail in the absence of individual splicing factors [46]. Although the mechanism of Ecd action remains to be determined, we presume that *spok* pre-mRNA might be particularly sensitive to the absence of Ecd, Prp8 or Brr2 for similar reasons.

### Ecdysone-dependent versus organ-autonomous consequences of Ecd depletion

In addition to abolishing the expression of Spok, loss of *ecd* function partially reduced mRNA and pre-mRNA levels of *phm* (Figure 6C, 6D) and of two other genes (*nvd* and *dib*; data not shown) that are likewise required for E biosynthesis. However, the lower expression of *phm*, *nvd* and *dib* was not accompanied by increased pre-mRNA:mRNA ratios, indicating that pre-mRNA splicing of these genes did not depend on Ecd. These results might suggest that Ecd exerts some effect on transcription or transcript stability. Alternatively, Ecd might be required for pre-mRNA splicing of a factor acting upstream of the steroidogenic gene expression. One such candidate is EcR, which has recently been shown to mediate a positive-feedback of 20E on the expression of *phm*, *dib*, *sro* and *sad*, but not of *spok* [49]. Elevated pre-mRNA:mRNA ratio indicated that splicing of *EcR* pre-mRNA was compromised in *ecd^1^* mutants (Figure 7H), and the EcR protein was markedly reduced in the PGs of *phm>ecd^RNAi^* larvae (Figure S6D). Therefore, whereas the absence of the Spok protein primarily resulted from disrupted splicing, the partial reduction of *nvd*, *phm* and *dib* expression might be attributable to the lack of EcR in the PG of *phm>ecd^RNAi^* larvae.

A failure of the PG to produce E inevitably provokes systemic developmental defects in peripheral organs. However, an issue arises with the *ecd^1^* mutants as to whether any observed tissue-specific phenotype may be ascribed to the lacking hormone. Because Ecd is required cell-autonomously, effects of ecdysteroid deficiency and those caused by loss of any cell-autonomous Ecd function cannot be discriminated in *ecd^1^* mutant background. For example, low expression of 20E-response genes such as *EcR* in *ecd^1^* larvae likely results from a combined impact of disrupted E synthesis in the PG and compromised *EcR* pre-mRNA splicing in the peripheral tissues. The problem of 20E-dependent and 20E-independent effects of the *ecd^1^* mutation may be reflected by a recent transcriptome analysis, revealing that of about a thousand genes affected in *ecd^1^* background, only a minority were regulated by the 20E receptor, EcR [43].

Based on the elimination of *ecd^l(3)23^* mutant clones from the imaginal discs, we conclude that cells lacking Ecd cannot be sustained within proliferating tissue context. This was the case even when the mutant clones were protected from apoptosis with p35, or enhanced for growth through Ras^V12^. Small *ecd^l(3)23^* or *ecd^RNAi^* clones were replaced by surrounding cells without phenotypic consequences for the adult. In contrast, *ecd* RNAi delivered to larger areas was lethal or caused visible defects, such as the aberrant wings in *dpp>ecd^RNAi^* flies. Rarely emerging *MS1096>ecd^RNAi^* flies had vestigial wings (Figure S7A) similar to those induced by depletion of the BCAS2 spliceosomal protein under the same Gal4 driver [25]. Interestingly, imaginal disc cells were not affected by triggering *ecd* RNAi after their proliferative phase was completed, as in the eyes of *GMR>ecd^RNAi^* flies (Figure S7B). *GMR-Gal4* is a strong but late-acting driver expressed predominantly in the post-mitotic cells posterior to the eye morphogenetic furrow [50]. Intriguingly, deletion of Ecd in mouse embryonic fibroblasts has been shown to cause a proliferative block and to reduce expression of several cell-cycle regulators such as CyclinE1 (CycE1) downstream of the transcription factor E2F [14]. However, we were unable to advance proliferation of *ecd^RNAi^* clones in imaginal discs by expressing *Drosophila* CycE [51] or combinations of either E2F with its partner DP [52] or CycD with Cdk4 [53] (data not shown). Therefore, the lack of Ecd in proliferating tissues cannot be compensated by gain of individual factors that stimulate cellular growth or cell cycle progression. These results suggest that proliferating cells are particularly sensitive to loss of Ecd. Such sensitivity corresponds to the fact that splicing factors prevailed among genes positively screened as being required for cell division in a human cell line [54].

### Evolving Ecd function?

Unlike some of the core spliceosomal factors, no Ecd-like protein has been found in the budding yeast, *S. cerevisiae*, in which only 5% of the genes contain introns. This contrasts with 43% of intron-containing genes in the fission yeast, *Schizosaccharomyces pombe* [55], where the spliceosomal protein composition is more akin to humans than to the budding yeast [56]. Interestingly, *S. pombe* has an Ecd ortholog, Sgt1p, which resides in the cell nuclei and is essential for the yeast growth [57]. Conditional *Sgt1* mutation alters expression of genes involved in virtually all cellular processes including metabolism, and impairs growth on glucose media. Sgt1p is thought to regulate transcription [57], although its mode of action remains unknown. Expression of Sgt1p failed to substitute for *Drosophila* Ecd (data not shown), likely reflecting a remote homology between the two proteins (21% overall amino acid identity). In contrast, human Ecd that is moderately homologous to fly Ecd (31%) rectified pre-mRNA splicing and expression of *spok* in the PG, permitting development of *phm>ecd^RNAi^* adults.

In mammals, Ecd has been implicated as a positive regulator of cell-cycle promoting genes, of cell cycle progression itself, and of cancer development [14]–[16]. Since hEcd has been independently detected in a complex containing human Prp8 [19], it will be of interest to know if any of those effects involve splicing of particular pre-mRNAs. Considering the evidence from *S. pombe*, *Drosophila* and mammals, we suspect that the role of Ecd in pre-mRNA splicing may correlate with the evolutionarily growing importance of splicing in complex multicellular organisms.

## Materials and Methods

### Fly stocks

The following *Drosophila* strains were used: *w^1118^*, *ecd^1^* [11], *ecd^l(3)23^* [13]; a gift of Dr I. Zhimulev), *FRT2A* (BL1997), *ecd^l(3)23^ FRT2A/TM6B*, *y w hsFLP; ecd^l(3)23^ FRT2A/TM6B*, *UAS-ras^V12^/CyO; ecd^l(3)23^ FRT2A/TM6B*, *UAS-p35/CyO; ecd^l(3)23^ FRT2A/TM6B*, *UAS-prp8^RNAi^* (VDRC, 18565), *UAS-brr2^RNAi^* (VDRC, 110666), *phm-Gal4, UAS-CD8::GFP* [33], and *dpp-Gal4, UAS-GFP* [58]. New transgenic lines carrying *UAS* **-** *ecd*, *UAS-ecd^RNAi^*, *UAS-Flag::hEcd*, *UAS-Flag::prp8*, and *UAS-Flag::Sgt1* constructs were established with standard *P*-element germline transformation. If not specified otherwise, crosses were carried out at 25°C. Crossing all Gal4 driver lines to *w^1118^* background provided controls for each experiment. Mutant clones within eye/antennal imaginal discs were generated using the MARCM (mosaic analysis with a repressible cell marker) method [32] with *eyFLP, act>y^+^>Gal4, UAS-GFP/CyO; FRT2A tubGal80/TM6B* flies as described [59]. To induce *ecd^RNAi^* “flip-out” clones, *hsFLP; act>y^+^>Gal4, UAS-GFP/CyO* [60] females were crossed to *UAS-ecd^RNAi^* males at 20°C. Recombination was induced by exposing progeny to heat shock at 37°C for 30 min, followed by incubation for 48 and 72 h at 25°C prior to dissection. Temperature-sensitive *ecd^1^* and control *w^1118^* larvae were grown at 22°C until day 4 AEL, then placed to 37°C for 45 min and to 29°C until dissection on day 6 AEL.

### Plasmids and Ecd antibody production

Coding *Drosophila melanogaster* DNA sequences of *prp8* (*CG8877*), *snu114* (*CG4849*), *aar2* (*CG12320*), *brr2* (*CG5931*), *ecd* (*CG5714*), *ecd^l(3)23^* (amino acids 1–650 of Ecd), and human *Ecd* (*hsgt1*, *NP_009196.1*) were amplified from respective cDNAs using the Phusion polymerase (New England Biolabs). *Schizosaccharomyces pombe Sgt1* (*SPAC1002.10c*) was amplified from genomic DNA. See Table S1 for all PCR primers. The fragments were cloned into pTFW, pTMW or pTGW vectors enabling expression of proteins with N-terminal Flag, Myc, or GFP tags, respectively (T. Murphy, Carnegie Institution of Washington), using the Gateway cloning system (Invitrogen). The *ecd^1^* P656S mutant was recreated using site-directed mutagenesis by CCT to TCT codon transition. To generate *UAS-ecd^RNAi^*, a 497-bp cDNA fragment from the *ecd* gene was amplified and cloned as inverted repeat into the pWIZ vector [61]. A polyclonal rat anti-Ecd^Nterm^ antibody was raised (Eurogentec) against bacterially expressed *Drosophila* Ecd polypeptide (amino acids 204–458). The corresponding *ecd* cDNA fragment was cloned into the pET28b plasmid (Novagen), and the antigen was purified using a hexahistidine tag.

### *Drosophila* S2 cell culture and immunoprecipitation

*Drosophila* S2 cells were cultured at 25°C in Shields and Sang M3 insect medium (Sigma Aldrich) containing 8% fetal bovine serum and antibiotics (Pen/Strep, Gibco). Cells were transfected in serum free medium using X-TremeGENE (Roche Applied Science) according to manufacturer's instructions. Expression of UAS-driven genes was induced by co-transfection with a pWA-GAL4 plasmid expressing Gal4 under an *actin5C* promoter (a gift from Y. Hiromi). Nuclear export was inhibited by incubating cells with 5 ng/ml leptomycin B (Biomol) for 4 h. Cells expressing the temperature-sensitive Ecd^1^ protein variant were upshifted to non-permissive temperature of 30°C for 45 min prior to processing.

For immunoprecipitation, transfected S2 cells were lysed in 50 mM Tris-HCl (pH 7.8), 150 mM NaCl, 1 mM EDTA (pH 8.0), 1% Triton X-100, 0.01% Igepal, and protease inhibitors (Roche Applied Science). The lysate (300–500 µg of total protein) was incubated overnight with 15 µl of anti-Flag (Invitrogen) or anti-Myc (Medical and Biological Laboratories) magnetic beads at 4°C. After five washes in lysis buffer, proteins were recovered in two consecutive elution steps, each with 50 µl of 0.1 M glycine-HCl (pH 3.0) for 5 min, and neutralized with 10 µl of 0.5 M Tris-HCl (pH 7.8) and 1.5 M NaCl. Upon SDS-PAGE, proteins were detected by immunoblotting with mouse anti-Flag M2 (1∶1000, Sigma Aldrich), rabbit anti-c-Myc (1∶1000, sc-789, Santa Cruz) or rabbit anti-GFP (1∶2000, Acris) antibodies, followed by incubation with corresponding HRP-conjugated secondary antibodies. Chemiluminescent signal was captured using ImageQuant LAS4000 reader (GE Healthcare).

### Mass spectrometry and protein identification

Protein extracts from S2 cells containing Myc::Ecd or the empty pTMW vector (for control) were resolved on SDS-PAGE. Upon silver staining (SilverQuestTM, Invitrogen), bands of interest were cut out. In-gel tryptic digestion with 12.5 ng/µl porcine trypsin (Promega) in 10 mM NH_4_HCO_3_ and further extractions were performed as described [62]. Collected extracts were concentrated by vacuum centrifugation and desalted using STAGE Tip C18 spin columns (Proxeon, Thermo Scientific) [63]. Eluted peptides were vacuum-concentrated and resuspended to a final volume of 20 µl in 0.5% acetic acid, of which 10 µl were used for analysis. Reversed-phase liquid chromatography (LC) coupled to nano-flow electrospray tandem mass spectrometry (MS) were carried out using an EASY nLC II nano-LC (Proxeon, Thermo Scientific) with a C18 column (internal diameter 75 µm) coupled to a LTQ Orbitrap mass spectrometer (Thermo Scientific). Peptide separation was performed at a flow rate of 250 nl/min over 60 min (10–40% acetonitrile; buffer A: 0.1% formic acid in water; buffer B: 0.1% formic acid in acetonitrile). Survey full scan MS spectra (m/z 350 to 2000) of intact peptides were acquired in the Orbitrap at a resolution of 30,000 using m/z 445.12003 as a lock mass. The mass spectrometer acquired spectra in data dependent mode and automatically switched between MS and MS/MS acquisition. Signals with unknown charge state and +1 were excluded from fragmentation. The ten most intense peaks (threshold 500) were isolated and automatically fragmented in the linear ion trap using collision induced dissociation (CID).

The search algorithm Mascot [64], implemented in the Proteinscape software (Bruker), was used for peptide and protein identification. MS/MS data were searched using the canonical and isoform sequence database of the *Drosophila melanogaster* complete proteome, provided by the UniProt Consortium. Oxidation of methionine residues was used as a variable modification and carbamidomethylation of cysteine residues as a fixed modification. For Orbitrap data, 10 ppm mass tolerance was allowed for intact peptide masses and 0.8 Da for CID fragment ions detected in the linear ion trap. Peptides were filtered for Mascot score ≤20. Protein identifications were based on at least two peptides.

### Cell and tissue immunostaining

*Drosophila* S2 cells grown on cover slips, dissected ring glands and imaginal discs were processed as described [65] and stained overnight at 4°C (tissues) or 2 h at room temperature (S2 cells) with the following antibodies: rat anti-Ecd^Nterm^ (1∶500, this study), guinea pig anti-Spok (1∶1000) and rabbit anti-Phm (1∶300) [33], rabbit anti-cleaved Caspase-3 (1∶500, ASP175, Cell Signaling, #9661), rabbit anti-pH3 (1∶100, Cell Signaling, #9701), mouse anti-Flag M2 (1∶500, Sigma Aldrich), rabbit anti-c-Myc (1∶500, sc-789, Santa Cruz), and mouse anti-Lamin (1∶500, ADL67.10), mouse anti-EcR (1∶200, DDA2.7), and mouse anti-Fasciclin III (1∶300); the latter three from the Developmental Studies Hybridoma Bank (DSHB, Iowa). After washing, samples were incubated with corresponding secondary antibodies coupled to Cy3 or Cy5 (Jackson ImmunoResearch), counterstained with DAPI (0.5 µg/ml, Invitrogen) to visualize nuclei, and mounted in Dabco:Mowiol (Sigma-Aldrich).

### Image acquisition and processing

Confocal single sections and stacks were acquired at room temperature with Olympus FV1000 confocal microscope. Maximum projections were generated using Fluoview 2.1c Software (Olympus) and ImageJ [66]. Final image processing including panel assembly, brightness and contrast adjustment were done in Photoshop CS5.1 (Adobe Systems, Inc.). To allow comparison among genotypes, images were taken and processed with the same settings. Adult wings were mounted in Hoyer's medium. Images of larvae, flies, adult wings, and Z-stacks of adult eyes were taken using Leica M165 FC fluorescent stereomicroscope equipped with a DFC490 CCD camera. Images were processed using a Multifocus module of the LAS 3.7.0 software (Leica).

### qRT-PCR

Total RNA was isolated from eight third-instar larvae with Isol-RNA Lysis Reagent (5 Prime). cDNA was synthesized from 2 µg of RNA treated with DNase I (Ambion) using random primers and Superscript III reverse transcriptase (Invitrogen, Carlsbad, CA). PCR was performed in triplicates with the SYBR green mix (Bio-Rad, Hercules, CA) using the CFX96 (Bio-Rad, Hercules, CA) or the 7900HT (Applied Biosystems) real-time PCR systems. qRT-PCR primers (Table S1) were designed to anneal at 62°C. All primers were initially tested by qPCR on serially diluted DNA templates and those deviating from typical standard curves were redesigned. Data were normalized to *rp49* transcript levels and fold changes were calculated using the ΔΔCT method [67] or the Relative standard curve method [68]. At least four biological replicates were analyzed per each experiment.

### Hormone feeding and ecdysteroid measurement

For hormone feeding, 40 larvae aged 5 days AEL were transferred per vial on food containing 100 µg/ml of 20E. Control food contained solvent only (6% w/w ethanol). Total RNA was isolated after 4 h (for *ecd^1^*) or one day of feeding (6 days AEL for *phm-Gal4* experiments); wandering behavior was photographed on day 7 AEL. To measure ecdysteroid titer, larvae 6 days AEL (15 animals per assay) were homogenized in 500 µl of methanol and centrifuged at 20,000 *g*. The pellets were re-extracted with 300 µl methanol and supernatants pooled and vacuum dried. The dried extracts were processed using the EIA immunoassay system (Cayman Chemical) as described [69]. Ecdysteroid concentration was calculated from an eight-point standard curve of serially diluted 20E.

### Statistical analyses

An unpaired two-tailed Student's *t*-test with unequal variation and one-way analysis of variance (ANOVA) with a post hoc Newman-Keuls Multiple Comparison Test were used to determine statistical significance for changes in gene expression in all qRT-PCR experiments, larval size, and ecdysteroid measurements.

## Supporting Information

Figure S1 Localization of Prp8 and Ecd, and RNAi-mediated depletion of Ecd in PG cells. Flag::Prp8 expressed under the *phm-Gal4* driver localizes to the PG cytoplasm (A). Endogenous (B) or overexpressed (D) Ecd protein primarily resides in the cytoplasm of PG cells. *phm-Gal4* driven RNAi silencing of *ecd* results in depletion of the endogenous Ecd protein specifically in the PG part of the ring gland. Ecd was visualized with an antibody against the N-terminal half of the *Drosophila* Ecd protein (Ecd^Nterm^; this study). Membrane-targeted CD8::GFP marks the PG cells, DAPI stains the nuclei, and anti-Lamin staining outlines the nuclear envelope. Panels show single confocal sections. (A′–D′) are magnified views of the PG cells. Scale bars, 20 µm (A–D), 5 µm (A′–D′).
(PDF)

Figure S2 Efficiency of Ecd RNAi knockdown in wing imaginal discs. Compared to control (A), *dpp-Gal4* driven expression of *UAS-ecd^RNAi^* (*dpp>ecd^RNAi^*) (B) results in marked depletion of endogenous Ecd protein in the cells along the anterior-posterior boundary (arrowheads) of third-instar wing discs. The *dpp* expression domain is visualized by co-expression of *UAS-GFP*. Note the cytoplasmic localization of Ecd. DAPI stains cell nuclei. Scale bars, 50 µm.
(PDF)

Figure S3 Brr2 is required for survival of imaginal cells; ectopic Prp8 does not rescue defects caused by *ecd* RNAi. (A) RNAi knockdown of Brr2 under the *dpp-Gal4* driver causes massive cell death of wing imaginal disc cells as visualized by staining with anti-cleaved Caspase 3 antibody (A′, arrows), and results in morphological anomalies of adult wings (B), namely loss of anterior crossvein (arrowhead) and reduced size of intervein region (double arrow). These defects phenocopy depletion of Ecd or Prp8 (compare with Figure 4B, 4C, 4F, 4G) but cannot be suppressed by supplementing *ecd^RNAi^* cells with extra Prp8 protein (*dpp>ecd^RNAi^ prp8*) (C, D). Scale bars, 100 µm (A, C), 20 µm (A′, C′), and 1 mm (B, D).
(PDF)

Figure S4 Expression of Spok and Phm proteins in the PGs subjected to RNAi against Brr2, a component of the U5 snRNP complex. (A) Staining of a control PG dissected 6 days AEL with anti-Spok (A′) and anti-Phm (A″) antibodies. (B) PG-specific RNAi targeting of Brr2 abolished expression of Spok (B′) and reduced levels of Phm (B″) proteins. Brr2 knockdown also altered PG morphology and size. Panels show single confocal sections. Scale bars, 20 µm.
(PDF)

Figure S5 (A) DNAse-treated RNA samples used for cDNA synthesis were free of contaminating genomic DNA as determined by end-point PCR (34 cycles). Primer sets specific to an *EcR* intron and to exons of the *rp49* gene amplified bands only in cDNA and genomic DNA but not in RNA samples from larvae of the indicated genotypes. Note the increased size of the *rp49* PCR product in genomic DNA due inclusion of an intron positioned between the primers. (B) A representative example of amplification curves obtained by qRT-PCR on *ecd^1^* and control cDNA samples with the *rp49* primer set shows that *rp49* mRNA level was not significantly altered by loss of *ecd* function and therefore was suitable for normalization of qRT-PCR data. The green line marks the amplification threshold (Ct value). (C) Expression of *α-tub84B* mRNA (normalized to *rp49*) did not change significantly between third-instar control (*w^1118^*) and *ecd^1^* larvae (all up-shifted to 29°C). Data are mean ± S.E.M; *n*≥4.
(PDF)

Figure S6 Systemic effects of Ecd deficiency. (A) Ecdysteroid content was reduced in whole *phm>ecd^RNAi^* larvae on day 6 AEL compared to controls (*w^1118^; phm-Gal4/+*), and it was increased above control levels by over-expression of hEcd (*phm>ecd^RNAi^ hEcd*). (B) After two days of feeding 20E, *phm>ecd^RNAi^* larvae (second from left), but not their solvent-treated siblings (far left), displayed wandering behavior on day 7 AEL. At that time, 20E-treated and untreated controls (*w^1118^; phm-Gal4/+*) began to pupariate. (C) Expression of *EcR* and *E74* genes (as assessed by qRT-PCR with primer sets detecting all alternatively spliced mRNA isoforms of each gene, see Table S1) were significantly higher in third-instar *ecd^1^* larvae reared at 22°C than in their siblings at the restrictive temperature (29°C) after 4 h of exposure to 20E. Levels of spliced *EcR* and *E74* mRNAs (all isoforms) were induced on day 6 AEL in *phm>ecd^RNAi^* larvae fed for 24 h on 20E-containing diet relative to controls of the same age. Note that the induction was weaker in *ecd^1^* mutants under 29°C. (D) EcR protein (detected with an antibody against all EcR isoforms) was markedly diminished upon depletion of Ecd (*phm>ecd^RNAi^*) from the PG (dissected 6 days AEL). Panels show single confocal sections. Scale bars, 20 µm.
(PDF)

Figure S7 Ecd is required in proliferating wing disc epithelium but dispensable in postmitotic imaginal cells of the developing eye. (A) *ecd* RNAi induced over an extended area of wing imaginal discs under the *MS1096-Gal4* driver yielded few adult escapers with a vestigial wing phenotype. (B) Adult flies emerged with externally normal eyes upon *GMR>ecd^RNAi^* targeting of cells posterior to the morphogenetic furrow, even when *dicer2* was co-expressed in order to enhance RNAi efficiency.
(PDF)

Table S1 List of primers used for expression constructs and qRT–PCR.
(PDF)

## References

[1] Thummel CS (2001) Molecular mechanisms of developmental timing in C. elegans and Drosophila. Dev Cell 1: 453–465. 11703937 10.1016/s1534-5807(01)00060-0

[2] Mirth CK, Riddiford LM (2007) Size assessment and growth control: how adult size is determined in insects. Bioessays 29: 344–355. 17373657 10.1002/bies.20552

[3] Yamanaka N, Rewitz KF, O'Connor MB (2013) Ecdysone control of developmental transitions: lessons from Drosophila research. Annu Rev Entomol 58: 497–516. 23072462 10.1146/annurev-ento-120811-153608 PMC4060523

[4] Ou Q, King-Jones K (2013) What goes up must come down: transcription factors have their say in making ecdysone pulses. Curr Top Dev Biol 103: 35–71. 23347515 10.1016/B978-0-12-385979-2.00002-2

[5] Rewitz KF, Yamanaka N, O'Connor MB (2013) Developmental checkpoints and feedback circuits time insect maturation. Curr Top Dev Biol 103: 1–33. 23347514 10.1016/B978-0-12-385979-2.00001-0 PMC4060521

[6] Yoshiyama-Yanagawa T, Enya S, Shimada-Niwa Y, Yaguchi S, Haramoto Y, et al (2011) The conserved Rieske oxygenase DAF-36/Neverland is a novel cholesterol-metabolizing enzyme. J Biol Chem 286: 25756–25762. 21632547 10.1074/jbc.M111.244384 PMC3138242

[7] Niwa R, Namiki T, Ito K, Shimada-Niwa Y, Kiuchi M, et al (2010) Non-molting glossy/shroud encodes a short-chain dehydrogenase/reductase that functions in the “Black Box” of the ecdysteroid biosynthesis pathway. Development 137: 1991–1999. 20501590 10.1242/dev.045641

[8] Huang X, Warren JT, Gilbert LI (2008) New players in the regulation of ecdysone biosynthesis. J Genet Genomics 35: 1–10. 18222403 10.1016/S1673-8527(08)60001-6

[9] Iga M, Kataoka H (2012) Recent studies on insect hormone metabolic pathways mediated by cytochrome P450 enzymes. Biol Pharm Bull 35: 838–843. 22687472 10.1248/bpb.35.838

[10] Petryk A, Warren JT, Marqués G, Jarcho MP, Gilbert LI, et al (2003) Shade is the Drosophila P450 enzyme that mediates the hydroxylation of ecdysone to the steroid insect molting hormone 20-hydroxyecdysone. Proc Natl Acad Sci USA 100: 13773–13778. 14610274 10.1073/pnas.2336088100 PMC283497

[11] Garen A, Kauvar L, Lepesant JA (1977) Roles of ecdysone in Drosophila development. Proc Natl Acad Sci USA 74: 5099–5103. 16592466 10.1073/pnas.74.11.5099 PMC432107

[12] Henrich VC, Tucker RL, Maroni G, Gilbert LI (1987) The ecdysoneless (ecd1ts) mutation disrupts ecdysteroid synthesis autonomously in the ring gland of Drosophila melanogaster. Dev Biol 120: 50–55. 3102296 10.1016/0012-1606(87)90102-3

[13] Gaziova I, Bonnette PC, Henrich VC, Jindra M (2004) Cell-autonomous roles of the ecdysoneless gene in Drosophila development and oogenesis. Development 131: 2715–2725. 15128659 10.1242/dev.01143

[14] Kim JH, Gurumurthy CB, Naramura M, Zhang Y, Dudley AT, et al (2009) Role of mammalian Ecdysoneless in cell cycle regulation. J Biol Chem 284: 26402–26410. 19640839 10.1074/jbc.M109.030551 PMC2785328

[15] Zhao X, Mirza S, Alshareeda A, Zhang Y, Gurumurthy CB, et al (2012) Overexpression of a novel cell cycle regulator ecdysoneless in breast cancer: a marker of poor prognosis in HER2/neu-overexpressing breast cancer patients. Breast Cancer Res Treat 134: 171–180. 22270930 10.1007/s10549-011-1946-8 PMC3397230

[16] Dey P, Rachagani S, Chakraborty S, Singh PK, Zhao X, et al (2012) Overexpression of ecdysoneless in pancreatic cancer and its role in oncogenesis by regulating glycolysis. Clin Cancer Res 18: 6188–6198. 22977192 10.1158/1078-0432.CCR-12-1789 PMC3551465

[17] Guruharsha KG, Rual J-F, Zhai B, Mintseris J, Vaidya P, et al (2011) A protein complex network of Drosophila melanogaster. Cell 147: 690–703. 22036573 10.1016/j.cell.2011.08.047 PMC3319048

[18] Grainger RJ, Beggs JD (2005) Prp8 protein: at the heart of the spliceosome. RNA 11: 533–557. 15840809 10.1261/rna.2220705 PMC1370742

[19] Havugimana PC, Hart GT, Nepusz T, Yang H, Turinsky AL, et al (2012) A census of human soluble protein complexes. Cell 150: 1068–1081. 22939629 10.1016/j.cell.2012.08.011 PMC3477804

[20] Maniatis T, Reed R (2002) An extensive network of coupling among gene expression machines. Nature 416: 499–506. 11932736 10.1038/416499a

[21] Jurica MS, Moore MJ (2003) Pre-mRNA splicing: awash in a sea of proteins. Mol Cell 12: 5–14. 12887888 10.1016/s1097-2765(03)00270-3

[22] Wahl MC, Will CL, Lührmann R (2009) The spliceosome: design principles of a dynamic RNP machine. Cell 136: 701–718. 19239890 10.1016/j.cell.2009.02.009

[23] Will CL, Lührmann R (2011) Spliceosome structure and function. Cold Spring Harb Perspect Biol 3 ((7)) . pii: a003707. 10.1101/cshperspect.a003707 PMC3119917 21441581

[24] Andersen DS, Tapon N (2008) Drosophila MFAP1 is required for pre-mRNA processing and G2/M progression. J Biol Chem 283: 31256–31267. 18765666 10.1074/jbc.M803512200 PMC2662187

[25] Chen P-H, Lee C-I, Weng Y-T, Tarn W-Y, Tsao Y-P, et al (2013) BCAS2 is essential for Drosophila viability and functions in pre-mRNA splicing. RNA 19: 208–218. 23249746 10.1261/rna.034835.112 PMC3543084

[26] Coelho CMA, Kolevski B, Walker CD, Lavagi I, Shaw T, et al (2005) A genetic screen for dominant modifiers of a small-wing phenotype in Drosophila melanogaster identifies proteins involved in splicing and translation. Genetics 171: 597–614. 15998720 10.1534/genetics.105.045021 PMC1456774

[27] Ray P, Luo X, Rao EJ, Basha A, Woodruff EA, et al (2010) The splicing factor Prp31 is essential for photoreceptor development in Drosophila. Protein Cell 1: 267–274. 21203973 10.1007/s13238-010-0035-9 PMC4875087

[28] Herold N, Will CL, Wolf E, Kastner B, Urlaub H, et al (2009) Conservation of the protein composition and electron microscopy structure of Drosophila melanogaster and human spliceosomal complexes. Mol Cell Biol 29: 281–301. 18981222 10.1128/MCB.01415-08 PMC2612486

[29] Boon K-L, Grainger RJ, Ehsani P, Barrass JD, Auchynnikava T, et al (2007) prp8 mutations that cause human retinitis pigmentosa lead to a U5 snRNP maturation defect in yeast. Nat Struct Mol Biol 14: 1077–1083. 17934474 10.1038/nsmb1303 PMC2584834

[30] Weber G, Cristão VF, de L Alves F, Santos KF, Holton N, et al (2011) Mechanism for Aar2p function as a U5 snRNP assembly factor. Genes Dev 25: 1601–1612. 21764848 10.1101/gad.635911 PMC3182018

[31] Kim JH, Gurumurthy CB, Band H, Band V (2010) Biochemical characterization of human Ecdysoneless reveals a role in transcriptional regulation. Biol Chem 391: 9–19. 19919181 10.1515/BC.2010.004 PMC3902786

[32] Lee T, Luo L (2001) Mosaic analysis with a repressible cell marker (MARCM) for Drosophila neural development. Trends Neurosci 24: 251–254. 11311363 10.1016/s0166-2236(00)01791-4

[33] Ono H, Rewitz KF, Shinoda T, Itoyama K, Petryk A, et al (2006) Spook and Spookier code for stage-specific components of the ecdysone biosynthetic pathway in Diptera. Dev Biol 298: 555–570. 16949568 10.1016/j.ydbio.2006.07.023

[34] Warren JT, O'Connor MB, Gilbert LI (2009) Studies on the Black Box: incorporation of 3-oxo-7-dehydrocholesterol into ecdysteroids by Drosophila melanogaster and Manduca sexta. Insect Biochem Mol Biol 39: 677–687. 19699302 10.1016/j.ibmb.2009.08.004

[35] Listerman I, Sapra AK, Neugebauer KM (2006) Cotranscriptional coupling of splicing factor recruitment and precursor messenger RNA splicing in mammalian cells. Nat Struct Mol Biol 13: 815–822. 16921380 10.1038/nsmb1135

[36] Pleiss JA, Whitworth GB, Bergkessel M, Guthrie C (2007) Transcript specificity in yeast pre-mRNA splicing revealed by mutations in core spliceosomal components. PLoS Biol 5: e90. 17388687 10.1371/journal.pbio.0050090 PMC1831718

[37] Stanek D, Pridalová-Hnilicová J, Novotný I, Huranová M, Blazíková M, et al (2008) Spliceosomal small nuclear ribonucleoprotein particles repeatedly cycle through Cajal bodies. Mol Biol Cell 19: 2534–2543. 18367544 10.1091/mbc.E07-12-1259 PMC2397305

[38] McBrayer Z, Ono H, Shimell M, Parvy J-P, Beckstead RB, et al (2007) Prothoracicotropic hormone regulates developmental timing and body size in Drosophila. Dev Cell 13: 857–871. 18061567 10.1016/j.devcel.2007.11.003 PMC2359579

[39] Gibbens YY, Warren JT, Gilbert LI, O'Connor MB (2011) Neuroendocrine regulation of Drosophila metamorphosis requires TGFβ/Activin signaling. Development 138: 2693–2703. 21613324 10.1242/dev.063412 PMC3109597

[40] Warren JT, Bachmann JS, Dai JD, Gilbert LI (1996) Differential incorporation of cholesterol and cholesterol derivatives into ecdysteroids by the larval ring glands and adult ovaries of Drosophila melanogaster: a putative explanation for the l(3)ecd1 mutation. Insect Biochem Mol Biol 26: 931–943. 9014338 10.1016/s0965-1748(96)00059-8

[41] Redfern CPF, Bownes M (1983) Pleiotropic effects of the “ecdysoneless-1” mutation of Drosophila melanogaster. Mol Gen Genet 189: 432–440.

[42] Sliter TJ (1989) Imaginal disc-autonomous expression of a defect in sensory bristle patterning caused by the lethal(3)ecdysoneless1 (1(3)ecd1) mutation of Drosophila melanogaster. Development 106: 347–354. 2512110 10.1242/dev.106.2.347

[43] Davis MB, Li T (2013) Genomic analysis of the ecdysone steroid signal at metamorphosis onset using ecdysoneless and EcRnull Drosophila melanogaster mutants. Genes Genomics 35: 21–46. 23482860 10.1007/s13258-013-0061-0 PMC3585846

[44] Fong YW, Zhou Q (2001) Stimulatory effect of splicing factors on transcriptional elongation. Nature 414: 929–933. 11780068 10.1038/414929a

[45] Bieberstein NI, Carrillo Oesterreich F, Straube K, Neugebauer KM (2012) First exon length controls active chromatin signatures and transcription. Cell Rep 2: 62–68. 22840397 10.1016/j.celrep.2012.05.019

[46] Faustino NA, Cooper TA (2003) Pre-mRNA splicing and human disease. Genes Dev 17: 419–437. 12600935 10.1101/gad.1048803

[47] Park JW, Parisky K, Celotto AM, Reenan RA, Graveley BR (2004) Identification of alternative splicing regulators by RNA interference in Drosophila. Proc Natl Acad Sci USA 101: 15974–15979. 15492211 10.1073/pnas.0407004101 PMC528766

[48] Mordes D, Luo X, Kar A, Kuo D, Xu L, et al (2006) Pre-mRNA splicing and retinitis pigmentosa. Mol Vis 12: 1259–1271. 17110909 PMC2683577

[49] Moeller ME, Danielsen ET, Herder R, O'Connor MB, Rewitz KF (2013) Dynamic feedback circuits function as a switch for shaping a maturation– inducing steroid pulse in Drosophila. Development 140: 4730–4739. 24173800 10.1242/dev.099739 PMC3833430

[50] Hay BA, Maile R, Rubin GM (1997) P element insertion-dependent gene activation in the Drosophila eye. Proc Natl Acad Sci USA 94: 5195–5200. 9144214 10.1073/pnas.94.10.5195 PMC24655

[51] Du W, Xie JE, Dyson N (1996) Ectopic expression of dE2F and dDP induces cell proliferation and death in the Drosophila eye. EMBO J 15: 3684–3692. 8670872 PMC452020

[52] Duronio RJ, O'Farrell PH (1995) Developmental control of the G1 to S transition in Drosophila: cyclin E is a limiting downstream target of E2F. Genes Dev 9: 1456–1468. 7601350 10.1101/gad.9.12.1456

[53] Datar SA, Jacobs HW, la Cruz de AF, Lehner CF, Edgar BA (2000) The Drosophila cyclin D-Cdk4 complex promotes cellular growth. EMBO J 19: 4543–4554. 10970848 10.1093/emboj/19.17.4543 PMC302080

[54] Kittler R, Putz G, Pelletier L, Poser I, Heninger A-K, et al (2004) An endoribonuclease-prepared siRNA screen in human cells identifies genes essential for cell division. Nature 432: 1036–1040. 15616564 10.1038/nature03159

[55] Wood V, Gwilliam R, Rajandream M-A, Lyne M, Lyne R, et al (2002) The genome sequence of Schizosaccharomyces pombe. Nature 415: 871–880. 11859360 10.1038/nature724

[56] Käufer NF, Potashkin J (2000) Analysis of the splicing machinery in fission yeast: a comparison with budding yeast and mammals. Nucleic Acids Res 28: 3003–3010. 10931913 10.1093/nar/28.16.3003 PMC108416

[57] Kainou T, Shinzato T, Sasaki K, Mitsui Y, Giga-Hama Y, et al (2006) Spsgt1, a new essential gene of Schizosaccharomyces pombe, is involved in carbohydrate metabolism. Yeast 23: 35–53. 16408318 10.1002/yea.1336

[58] Takaesu NT, Johnson AN, Newfeld SJ (2002) Posterior spiracle specific GAL4 lines: new reagents for developmental biology and respiratory physiology. Genesis 34: 16–18. 12324940 10.1002/gene.10109

[59] Uhlirova M, Jasper H, Bohmann D (2005) Non-cell-autonomous induction of tissue overgrowth by JNK/Ras cooperation in a Drosophila tumor model. Proc Natl Acad Sci USA 102: 13123–13128. 16150723 10.1073/pnas.0504170102 PMC1201591

[60] Struhl G, Basler K (1993) Organizing activity of wingless protein in Drosophila. Cell 72: 527–540. 8440019 10.1016/0092-8674(93)90072-x

[61] Sik Lee Y, Carthew RW (2003) Making a better RNAi vector for Drosophila: use of intron spacers. Methods 30: 322–329. 12828946 10.1016/s1046-2023(03)00051-3

[62] Steinfeldt T, Könen-Waisman S, Tong L, Pawlowski N, Lamkemeyer T, et al (2010) Phosphorylation of mouse immunity-related GTPase (IRG) resistance proteins is an evasion strategy for virulent Toxoplasma gondii. PLoS Biol 8: e1000576. 21203588 10.1371/journal.pbio.1000576 PMC3006384

[63] Rappsilber J, Mann M, Ishihama Y (2007) Protocol for micro-purification, enrichment, pre-fractionation and storage of peptides for proteomics using StageTips. Nat Protoc 2: 1896–1906. 17703201 10.1038/nprot.2007.261

[64] Perkins DN, Pappin DJ, Creasy DM, Cottrell JS (1999) Probability-based protein identification by searching sequence databases using mass spectrometry data. Electrophoresis 20: 3551–3567. 10612281 10.1002/(SICI)1522-2683(19991201)20:18<3551::AID-ELPS3551>3.0.CO;2-2

[65] Külshammer E, Uhlirova M (2013) The actin cross-linker Filamin/Cheerio mediates tumor malignancy downstream of JNK signaling. J Cell Sci 126: 927–938. 23239028 10.1242/jcs.114462

[66] Abràmoff MD, Magalhães PJ, Ram SJ (2004) Image processing with ImageJ. Biophotonics International 11: 36–42.

[67] Livak KJ, Schmittgen TD (2001) Analysis of relative gene expression data using real-time quantitative PCR and the 2(-ΔΔC(T)) Method. Methods 25: 402–408. 11846609 10.1006/meth.2001.1262

[68] Larionov A, Krause A, Miller W (2005) A standard curve based method for relative real time PCR data processing. BMC Bioinformatics 6: 62. 15780134 10.1186/1471-2105-6-62 PMC1274258

[69] Ou Q, Magico A, King-Jones K (2011) Nuclear receptor DHR4 controls the timing of steroid hormone pulses during Drosophila development. PLoS Biol 9: e1001160. 21980261 10.1371/journal.pbio.1001160 PMC3181225

