# Supplementary figures and images for: Unexpected Role of the Steroid-Deficiency Protein Ecdysoneless in Pre-mRNA Splicing
Source: PLoS Genet. 2014 Apr 10;10(4):e1004287. doi: 10.1371/journal.pgen.1004287 (PMC3983036; doi:10.1371/journal.pgen.1004287)

**Fig. S1**

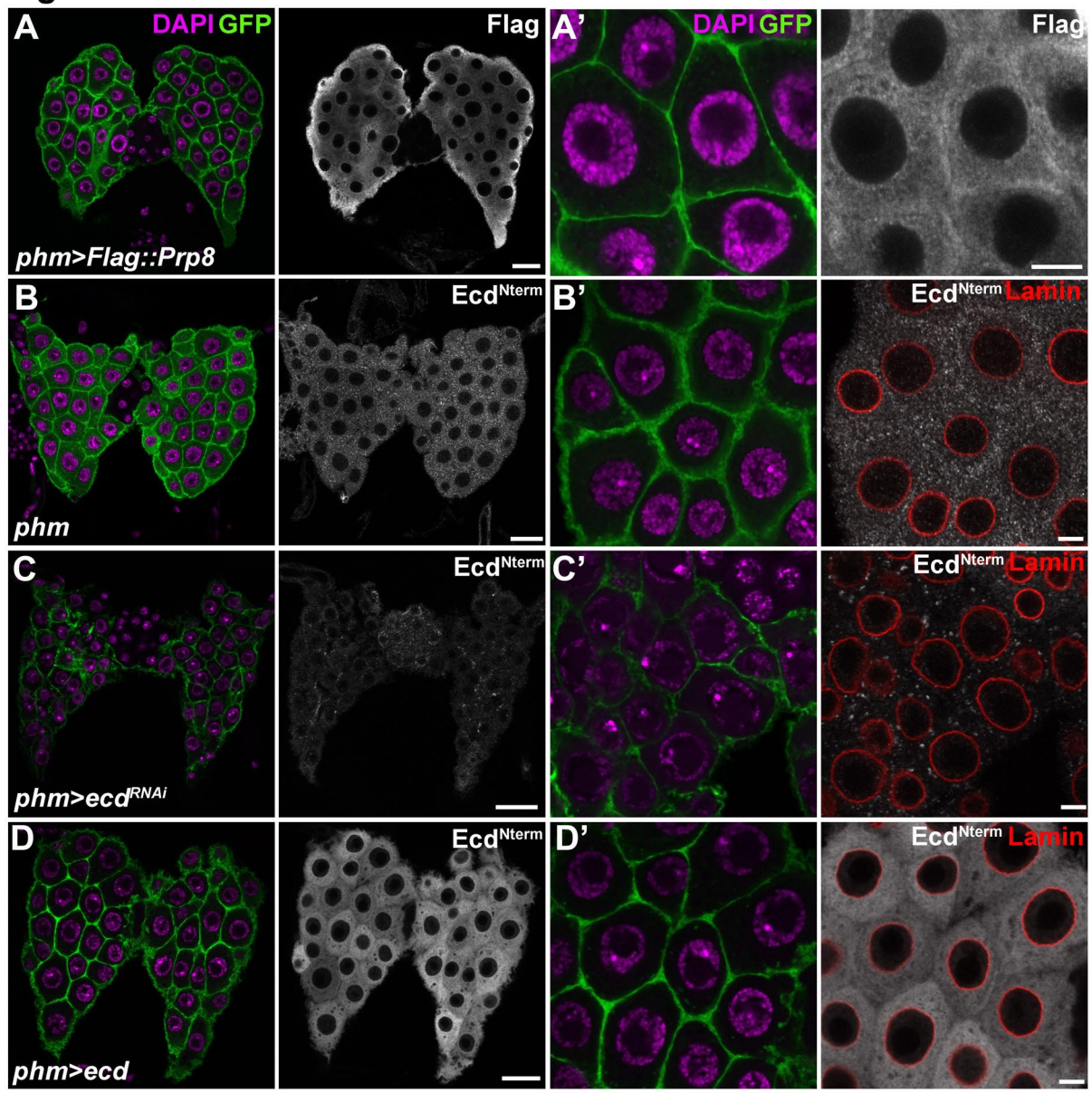

Supplement: Figure S1 — Localization of Prp8 and Ecd, and RNAi-mediated depletion of Ecd in PG cells. Flag::Prp8 expressed under the phm-Gal4 driver localizes to the PG cytoplasm (A). Endogenous (B) or overexpressed (D) Ecd protein primarily resides in the cytoplasm of PG cells. phm-Gal4 driven RNAi silencing of ecd results in depletion of the endogenous Ecd protein specifically in the PG part of the ring gland. Ecd was visualized with an antibody against the N-terminal half of the Drosophila Ecd protein (EcdNterm; this study). Membrane-targeted CD8::GFP marks the PG cells, DAPI stains the nuclei, and anti-Lamin staining outlines the nuclear envelope. Panels show single confocal sections. (A′–D′) are magnified views of the PG cells. Scale bars, 20 µm (A–D), 5 µm (A′–D′). (PDF) [file pgen.1004287.s001.pdf]

**Fig. S2**

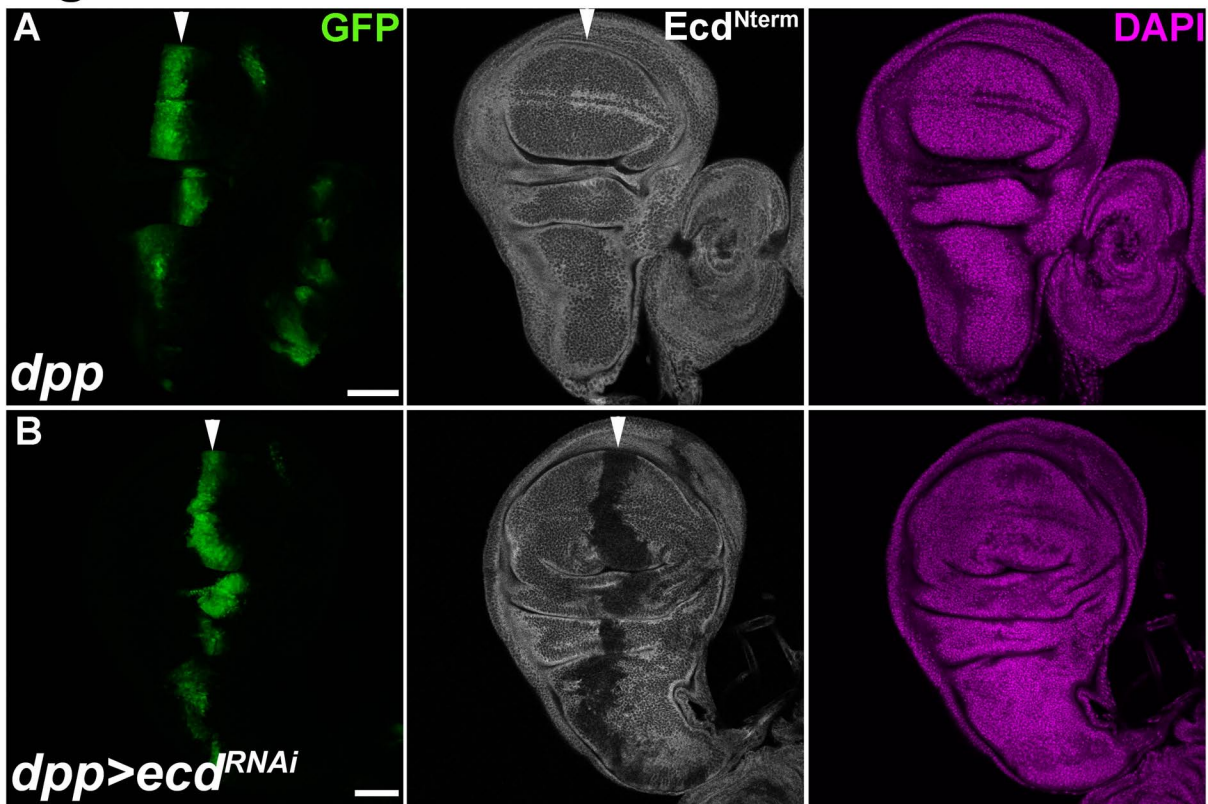

Supplement: Figure S2 — Efficiency of Ecd RNAi knockdown in wing imaginal discs. Compared to control (A), dpp-Gal4 driven expression of UAS-ecdRNAi (dpp>ecdRNAi) (B) results in marked depletion of endogenous Ecd protein in the cells along the anterior-posterior boundary (arrowheads) of third-instar wing discs. The dpp expression domain is visualized by co-expression of UAS-GFP. Note the cytoplasmic localization of Ecd. DAPI stains cell nuclei. Scale bars, 50 µm. (PDF) [file pgen.1004287.s002.pdf]

**Fig. S3**

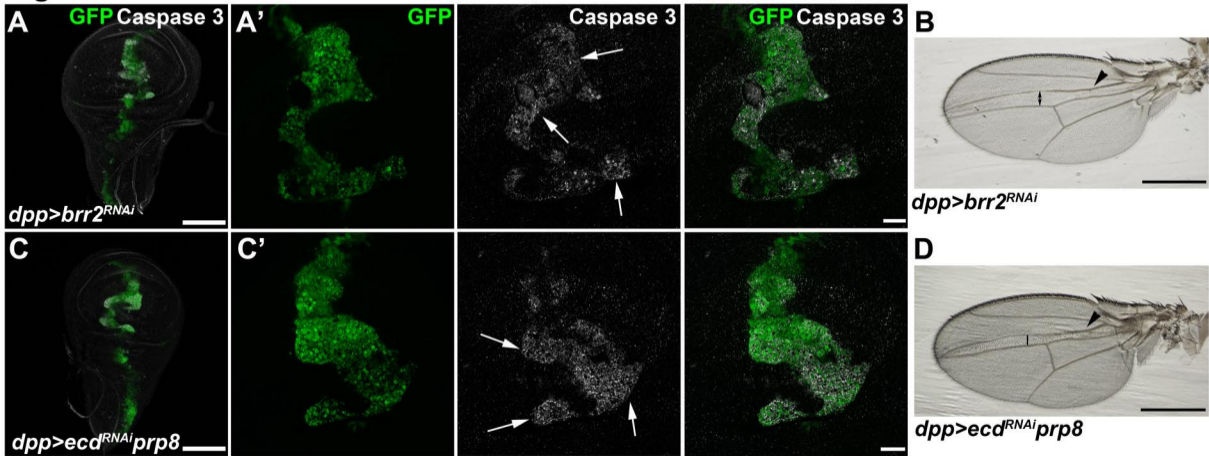

Supplement: Figure S3 — Brr2 is required for survival of imaginal cells; ectopic Prp8 does not rescue defects caused by ecd RNAi. (A) RNAi knockdown of Brr2 under the dpp-Gal4 driver causes massive cell death of wing imaginal disc cells as visualized by staining with anti-cleaved Caspase 3 antibody (A′, arrows), and results in morphological anomalies of adult wings (B), namely loss of anterior crossvein (arrowhead) and reduced size of intervein region (double arrow). These defects phenocopy depletion of Ecd or Prp8 (compare with Figure 4B, 4C, 4F, 4G) but cannot be suppressed by supplementing ecdRNAi cells with extra Prp8 protein (dpp>ecdRNAi prp8) (C, D). Scale bars, 100 µm (A, C), 20 µm (A′, C′), and 1 mm (B, D). (PDF) [file pgen.1004287.s003.pdf]

Fig. S4

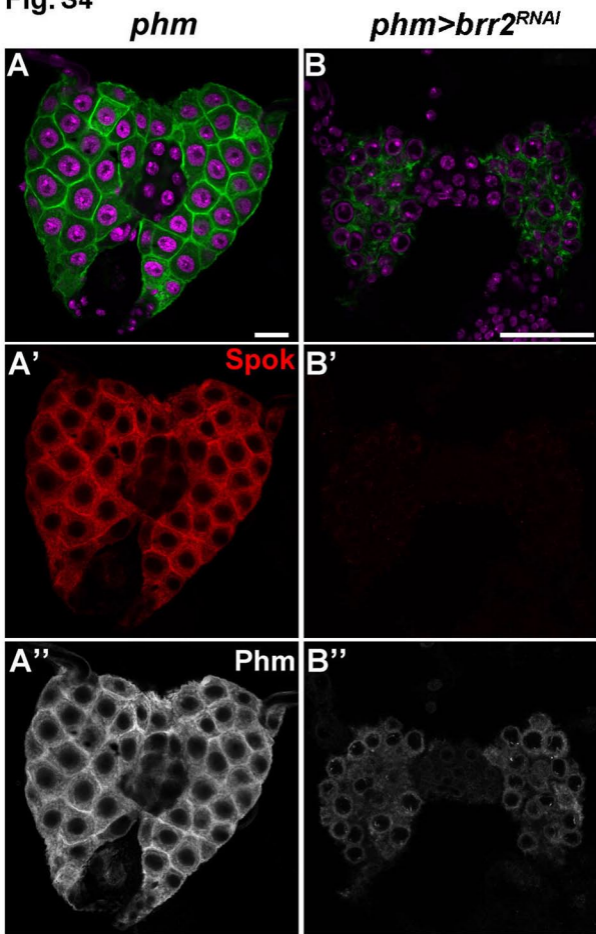

Supplement: Figure S4 — Expression of Spok and Phm proteins in the PGs subjected to RNAi against Brr2, a component of the U5 snRNP complex. (A) Staining of a control PG dissected 6 days AEL with anti-Spok (A′) and anti-Phm (A″) antibodies. (B) PG-specific RNAi targeting of Brr2 abolished expression of Spok (B′) and reduced levels of Phm (B″) proteins. Brr2 knockdown also altered PG morphology and size. Panels show single confocal sections. Scale bars, 20 µm. (PDF) [file pgen.1004287.s004.pdf]

**Fig. S5**  
**A**

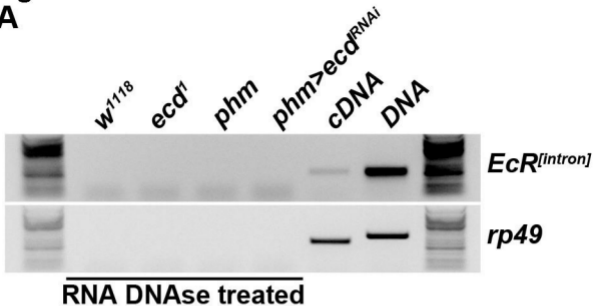

**B**

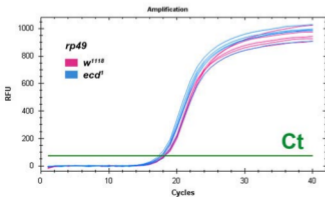

**C**

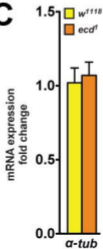

Supplement: Figure S5 — (A) DNAse-treated RNA samples used for cDNA synthesis were free of contaminating genomic DNA as determined by end-point PCR (34 cycles). Primer sets specific to an EcR intron and to exons of the rp49 gene amplified bands only in cDNA and genomic DNA but not in RNA samples from larvae of the indicated genotypes. Note the increased size of the rp49 PCR product in genomic DNA due inclusion of an intron positioned between the primers. (B) A representative example of amplification curves obtained by qRT-PCR on ecd1 and control cDNA samples with the rp49 primer set shows that rp49 mRNA level was not significantly altered by loss of ecd function and therefore was suitable for normalization of qRT-PCR data. The green line marks the amplification threshold (Ct value). (C) Expression of α-tub84B mRNA (normalized to rp49) did not change significantly between third-instar control (w1118) and ecd1 larvae (all up-shifted to 29°C). Data are mean ± S.E.M; n≥4. (PDF) [file pgen.1004287.s005.pdf]

Fig. S6

A

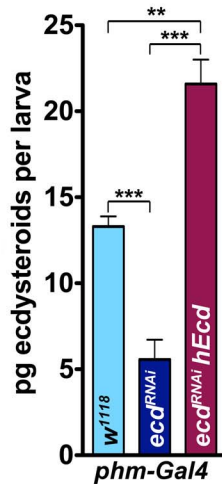

B

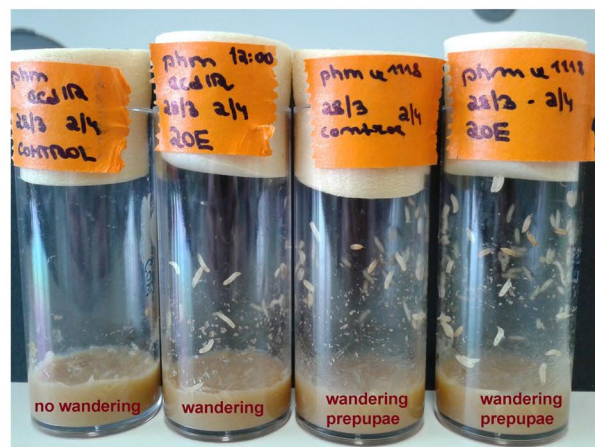

C

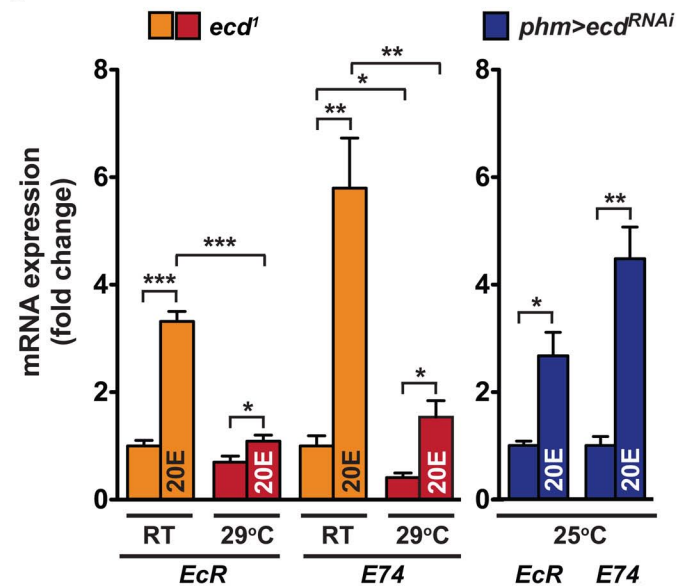

D

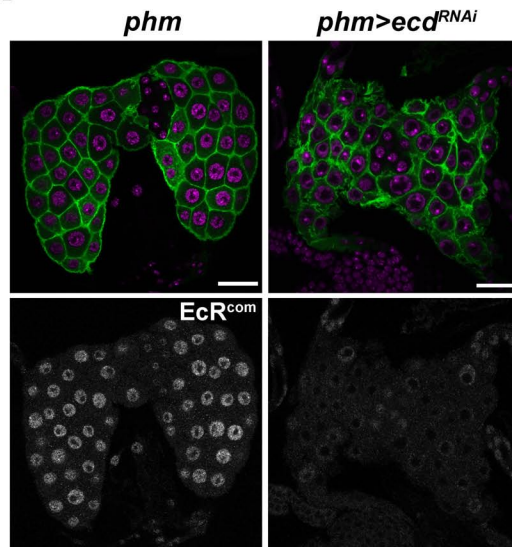

Supplement: Figure S6 — Systemic effects of Ecd deficiency. (A) Ecdysteroid content was reduced in whole phm>ecdRNAi larvae on day 6 AEL compared to controls (w1118; phm-Gal4/+), and it was increased above control levels by over-expression of hEcd (phm>ecdRNAi hEcd). (B) After two days of feeding 20E, phm>ecdRNAi larvae (second from left), but not their solvent-treated siblings (far left), displayed wandering behavior on day 7 AEL. At that time, 20E-treated and untreated controls (w1118; phm-Gal4/+) began to pupariate. (C) Expression of EcR and E74 genes (as assessed by qRT-PCR with primer sets detecting all alternatively spliced mRNA isoforms of each gene, see Table S1) were significantly higher in third-instar ecd1 larvae reared at 22°C than in their siblings at the restrictive temperature (29°C) after 4 h of exposure to 20E. Levels of spliced EcR and E74 mRNAs (all isoforms) were induced on day 6 AEL in phm>ecdRNAi larvae fed for 24 h on 20E-containing diet relative to controls of the same age. Note that the induction was weaker in ecd1 mutants under 29°C. (D) EcR protein (detected with an antibody against all EcR isoforms) was markedly diminished upon depletion of Ecd (phm>ecdRNAi) from the PG (dissected 6 days AEL). Panels show single confocal sections. Scale bars, 20 µm. (PDF) [file pgen.1004287.s006.pdf]

**Fig. S7**

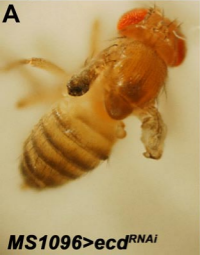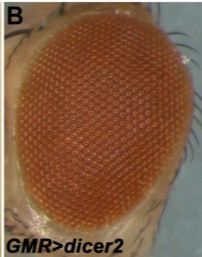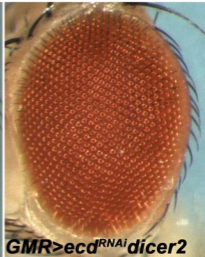

Supplement: Figure S7 — Ecd is required in proliferating wing disc epithelium but dispensable in postmitotic imaginal cells of the developing eye. (A) ecd RNAi induced over an extended area of wing imaginal discs under the MS1096-Gal4 driver yielded few adult escapers with a vestigial wing phenotype. (B) Adult flies emerged with externally normal eyes upon GMR>ecdRNAi targeting of cells posterior to the morphogenetic furrow, even when dicer2 was co-expressed in order to enhance RNAi efficiency. (PDF) [file pgen.1004287.s007.pdf]
