# Supplementary material for: Unexpected Role of the Steroid-Deficiency Protein Ecdysoneless in Pre-mRNA Splicing
Source: PLoS Genet. 2014 Apr 10;10(4):e1004287. doi: 10.1371/journal.pgen.1004287 (PMC3983036; doi:10.1371/journal.pgen.1004287)
Supplement: Table S1 — List of primers used for expression constructs and qRT–PCR. (PDF) [file pgen.1004287.s008.pdf]

Table S1

	Forward primers (5'=>3')	Reverse primers (5'=>3')
	**Expression constructs**	
ecd^{RNAi}	TTCGGCTTGTAGAGCTTCTTGGGT	TAATCTAGATTGTCGCACCAGCAGTATCTGCC<br>TAAGAATTCTTGTCGCACCAGCAGTATCTGCC
prp8	AAATGATCACGATGTCCATTCCGCCGTACATG	AAAGCGGCCGCGCTTACGCGTACACATCCTCCC
snu114	AAATGATCACCATGGATTCCGATTTGTACGATGA	AAAGCGGCCGCGCCTAAAGCGGGTAGTTGATCAGC
aar2	AAAGTCGACATGAACAAGGACAACTCCAGCA	AAACTCGAGCTAGGCTTCCGGAAGTTCGAT
brr2	CCCGTCTCAGATCCACATGGCGGATGCCGCAGCACG	AAAGCGGCCGCGCCTAATCTGACTCGCTTTCGCT
ecd	AAAGTCGACATGAGCAAGATTCCAGGCAGCA	ATATATCTGAGTCGGCCGTTTAAGCGGCCGCTT
ecd$^{l(3)23}$	AAAGTCGACATGAGCAAGATTCCAGGCAGCA	AAAGCGGCCGCTTAGTAGCTATCCATCATGTTGC
ecd^{1} mutagenesis	TGGCGGCGCAGGTTCTGTATCCAATC	GATTGGATACAGAACCTGCGCCGCCA
ecd^{Nterm} Ab production	AACATATGTCGCAGGAGATACAGTCGTGCAT	AACTCGAGCGGCTTGTAGAGCTTCTTGGGT
hEcd	AAAAGCGGCCGCATGGAAGAAACCATGAAGCT	AAAAGTCGACTTAATTTTTTGTTGGCTTACTTGT
sgt1	AAAAGCGGCCGCATGAGCAATCTAGGCAATAACATC	AAAACTCGAGTTGGAGGATATCATAATCTCATCAAAGG
	**qRT-PCR**	
rp49	TCCTACCAGCTTCAAGATGAC	CACGTTGTGCACCAGGAACT
spok	GCTCTTTGGCGGTGATCGAAACAA	CGCCGAGCTAAATTTCTCCGCTTT
spok intron	CGAGAATTTGGTTGCCAGCGAAACA	GCCATCCTCTTAAGGAGTGTGGTCAT
spok EI1	TCACAAGCTCTTTGCCGGTGAT	TTAAGAGGATGGATCTAGTGGCCC
spok EI2	CTTTCTTCTGCCTGTGAGATA	ACCTCCGATCTCGGACATCTT
phm	ATTGGCTAAAGGCCTTGGGCATGA	ATCCCGAAGACCAGGTCGTTGATT
phm intron	TGGCGCGTATTTACACTGAACG	ACGTAGATCCTGAAACCATACGA
ecd	ACCCAAGAAGCTCTACAAGCCGAA	ACCTTTGCTGTCAGTCCTGTGGAA
EcR^{com}	AAGGCCCAGAAGGAGAAGGACAAA	TATATTGCGCGCTTGACACTTGGC
EcR intron	TTAGGGTCGTCGGCTTTCGCTTAT	TTAGGCTAAGGGCTTGCTGTGACT
E74^{com}	GGGCACCTTTCTGCATCCGAATTT	GTGCGTTGAAGTAGGACGTTGTCA
E74 intron	CGGAGGCAACTTTCAATTGCGACA	TTACACAAGCACGCGAACACACAC
α-*tub84B*	TCATAGCCGGCAGTTCGAACGTAT	ACACCAGCCTGACCAACATGGATA
